# Broadly Neutralizing Antibody PGT121 Allosterically Modulates CD4 Binding via Recognition of the HIV-1 gp120 V3 Base and Multiple Surrounding Glycans

**DOI:** 10.1371/journal.ppat.1003342

**Published:** 2013-05-02

**Authors:** Jean-Philippe Julien, Devin Sok, Reza Khayat, Jeong Hyun Lee, Katie J. Doores, Laura M. Walker, Alejandra Ramos, Devan C. Diwanji, Robert Pejchal, Albert Cupo, Umesh Katpally, Rafael S. Depetris, Robyn L. Stanfield, Ryan McBride, Andre J. Marozsan, James C. Paulson, Rogier W. Sanders, John P. Moore, Dennis R. Burton, Pascal Poignard, Andrew B. Ward, Ian A. Wilson

**Affiliations:** 1 Department of Integrative Structural and Computational Biology, The Scripps Research Institute, La Jolla, California, United States of America; 2 IAVI Neutralizing Antibody Center, The Scripps Research Institute, La Jolla, California, United States of America; 3 CHAVI-ID, The Scripps Research Institute, La Jolla, California, United States of America; 4 Department of Immunology and Microbial Science, The Scripps Research Institute, La Jolla, California, United States of America; 5 Weill Medical College of Cornell University, New York, New York, United States of America; 6 Department of Chemical Physiology, The Scripps Research Institute, La Jolla, California, United States of America; 7 Department of Medical Microbiology, Academic Medical Center, Amsterdam, Netherlands; 8 Ragon Institute of MGH, MIT, and Harvard, Cambridge, Massachusetts, United States of America; 9 Skaggs Institute for Chemical Biology, The Scripps Research Institute, La Jolla, California, United States of America; University of Zurich, Switzerland

## Abstract

New broad and potent neutralizing HIV-1 antibodies have recently been described that are largely dependent on the gp120 N332 glycan for Env recognition. Members of the PGT121 family of antibodies, isolated from an African donor, neutralize ∼70% of circulating isolates with a median IC_50_ less than 0.05 µg ml^−1^. Here, we show that three family members, PGT121, PGT122 and PGT123, have very similar crystal structures. A long 24-residue HCDR3 divides the antibody binding site into two functional surfaces, consisting of an open face, formed by the heavy chain CDRs, and an elongated face, formed by LCDR1, LCDR3 and the tip of the HCDR3. Alanine scanning mutagenesis of the antibody paratope reveals a crucial role in neutralization for residues on the elongated face, whereas the open face, which accommodates a complex biantennary glycan in the PGT121 structure, appears to play a more secondary role. Negative-stain EM reconstructions of an engineered recombinant Env gp140 trimer (SOSIP.664) reveal that PGT122 interacts with the gp120 outer domain at a more vertical angle with respect to the top surface of the spike than the previously characterized antibody PGT128, which is also dependent on the N332 glycan. We then used ITC and FACS to demonstrate that the PGT121 antibodies inhibit CD4 binding to gp120 despite the epitope being distal from the CD4 binding site. Together, these structural, functional and biophysical results suggest that the PGT121 antibodies may interfere with Env receptor engagement by an allosteric mechanism in which key structural elements, such as the V3 base, the N332 oligomannose glycan and surrounding glycans, including a putative V1/V2 complex biantennary glycan, are conformationally constrained.

## Author Summary

An estimated 33 million adults and children currently live with the human immunodeficiency virus type 1 (HIV-1), which represents a global prevalence of 0.8%. In the absence of a cure, the development of a protective vaccine is the long sought-after goal in containment of the pandemic. HIV-1 Env is the sole viral surface glycoprotein and mediates viral engagement and entry into host cells, which constitutes the first step of the virus life cycle. Recently, a plethora of exciting new antibodies have been discovered that interact with HIV-1 Env and inhibit infection of target cells (i.e. neutralize the virus). Here, we structurally characterize the interaction of a recombinant HIV-1 Env with one class of such antibodies, namely antibodies of the PGT121 family. These studies have uncovered a novel mode of HIV-1 Env recognition. By interacting with key structural elements of HIV-1 Env near the apex at its membrane-distal end, these antibodies can interfere with binding to CD4, the receptor on T cells that is required for HIV-1 infection. These observations further delineate a glycan-dependent site of vulnerability on HIV-1 Env that can be used in vaccine design efforts.

## Introduction

The discovery of novel monoclonal antibodies capable of neutralizing a broad spectrum of HIV-1 isolates of various clades (broadly neutralizing antibodies, bnAbs) has rapidly accelerated in the past three years and provided valuable new reagents and opportunities for HIV vaccine design [1], [2], [3], [4], [5], [6], [7], [8], [9]. In other related fields, similar bursts of activity have identified new bnAbs to influenza and hepatitis C viruses [10], [11], [12], [13]. For HIV-1, screening of thousands of HIV-1 infected individuals in different cohorts has revealed that a relatively large proportion (approximately 20–25%) develop sera with moderate to broad neutralization characteristics that requires at least one to three years of infection, and that approximately 1% of infected individuals, termed elite neutralizers, develop an exceptionally broad and potent neutralizing antibody response [4], [14], [15], [16]. Fine-mapping of the broadly neutralizing activity of the elite neutralizers sera on the HIV-1 Env glycoprotein revealed five predominant neutralizing antibody specificities: 1) the CD4 binding site on gp120; 2) glycan-dependent epitopes on gp120 that are N332A sensitive; 3) an epitope in the vicinity of the CD4-induced site; 4) a quaternary epitope on gp120 that is sensitive to the loss of glycosylation at position N160; and 5) the conserved gp41 membrane proximal external region (MPER) [17], [18], [19], [20]. The number of bnAbs targeting these sites has increased exponentially in recent years due to the identification of suitable infected donors and the development of new technologies allowing for their identification and isolation [3], [5], [6], [7], [21], [22]. Together, structural and functional characterization of the vulnerable sites on HIV-1 Env, as well as the bnAbs that recognize them, are at the center of current vaccine development efforts.

Long thought as an impenetrable shield masking the conserved functional sites on HIV-1, the dense glycan coat of Env has emerged in recent years as an unexpected target for recognition by bnAbs. One specific area surrounding the base of the V3 loop on the outer domain of gp120 harbors a dense cluster of conserved oligomannose carbohydrates [23], [24], [25], [26]. 2G12, a bnAb capable of neutralizing 32% of a large panel of HIV-1 isolates at an IC_50_<50 µg/mL, was found several years ago to recognize an epitope in this region, namely the Manα1-2Man tips of certain oligomannose glycans. The N-linked glycans that have been implicated in the recognition by 2G12 are located at positions N295, N332, N339, N386, and N392 on the gp120 outer domain [5], [27], [28]. More recently, the crystal structure of PGT128 in complex with an engineered gp120 outer domain revealed that this bnAb achieves highly potent neutralization of about 70% of HIV-1 isolates by intimately interacting with two oligomannose glycans at positions N301 and N332, as well as with the base of the gp120 V3 loop [5], [29]. Together, these two bnAbs help to define more precisely the N332 site of vulnerability on HIV-1 Env.

Identified from African donor 17 of the IAVI Protocol G cohort, the PGT121 antibody family consists of three primary members (PGT121, PGT122 and PGT123), and additional antibodies from this donor described recently [30]. At an IC_50_<50 µg/mL, this family of bnAbs neutralizes 65–70% of HIV-1 isolates and also recognizes an N332-sensitive epitope [5]. Along with PGT128, members of the PGT121 family are the most potent anti-HIV-1 bnAbs identified to date, with a median IC_50_ ranging between 0.03–0.05 µg/mL [5]. However, the PGT121 family differs from the PGT128 family in that it is not as dependent on glycosylation at position N301 for most isolates and competes more extensively with PG9 binding, which recognizes a quaternary epitope composed principally of the gp120 V1/V2 loop and associated glycans [5]. These findings suggest a novel mechanism of recognition of the N332-sensitive gp120 outer domain epitope by the PGT121 family. Here, we describe the characterization of the PGT121 family at the atomic level by comparing crystal structures of all three family members. Paratope mapping and binding data have enabled characterization of the most crucial elements required for neutralization by this antibody family. Furthermore, we present a molecular-level characterization of the epitope recognized by PGT122 on a recombinant HIV-1 trimer (SOSIP.664) by electron microscopy at 15 Å resolution. Finally, biophysical experiments reveal that antibodies of the PGT121 family can interfere with gp120 binding to CD4, despite the PGT121 epitope being distally located from the CD4 binding site on gp120.

## Results

### Crystal structures of PGT121, 122 and 123 Fab

**Figure 1:**
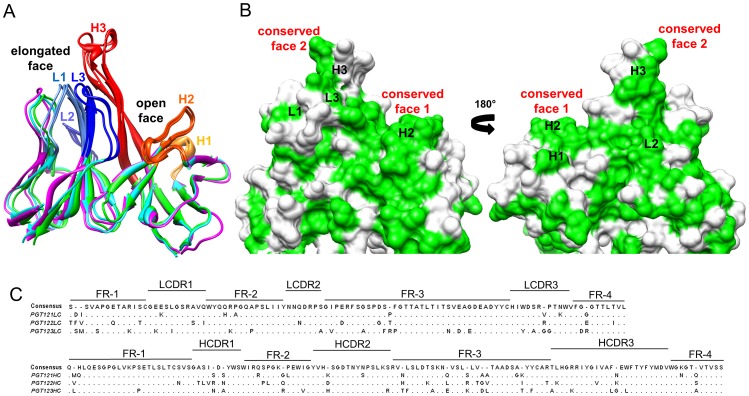
Structure and sequence characterization of antibodies of the PGT121 family. A) Superimposition of the PGT121 (green), PGT122 (cyan) and PGT123 (magenta) Fab crystal structures determined at 2.8 Å, 1.8 Å and 2.5 Å resolutions, respectively. Only the variable domains are shown with secondary structure rendering. The three CDRs of the light and heavy chains are labeled and colored in different shades of blue and red to orange, respectively. The 24-residue HCDR3 loop divides the paratope into two faces: an open face composed from HCDR1, HCDR2 and the base of HCDR3 and a more elongated face from LCDR1, LCDR3 and the tip of HCDR3. B) Sequence conservation between antibodies of the PGT121 family mapped on the PGT122 crystal structure, which is rendered as a surface representation. Identical residues in the three antibodies are colored green while divergent sequences are represented in white. Two regions of clustered sequence identity are predicted to play an important role for antigen recognition (conserved faces 1 and 2). These figures were generated using UCSF Chimera [70]. C) Sequence alignment among antibodies of the PGT121 family with the consensus sequence shown above and non-conserved residues shown below for each antibody. The six light chain (LC) and heavy chain (HC) CDRs and Framework Regions (FR) are indicated based on Kabat alignment. This figure was generated using Jalview [72].

To characterize the binding site, or paratope, for this new class of bnAbs at the atomic level, crystal structures of the PGT121, 122 and 123 Fabs were determined at resolutions of 2.8, 1.8 and 2.5 Å, respectively (Table 1). Strikingly, the overall structures of the three antibodies are highly similar, with root-mean-square deviation (r.m.s.d) values calculated for main-chain atoms ranging between 0.7 and 1.2 Å for the light and heavy chains of the Fv regions of all three antibodies (Fig. 1A), as well as with the previously described 10-1074 antibody of the same class [30]. One of the main characteristics of the paratope is a 24-residue HCDR3 that forms an extended anti-parallel β-hairpin with either a type-1 β-turn at its tip for PGT121 and PGT123 or a distorted 3_10_-helix at its tip for PGT122. This elongated HCDR3 divides the antibody recognition site into two faces (Fig. 1A). Residues from HCDR1 and HCDR2, as well as residues located at the base of HCDR3, form a U-shaped depression containing HCDR1 Tyr^H33^, HCDR2 Asp^H56^ and HCDR3 His^H97^ at its edges. These residues are conserved for all three antibodies and are accessible for forming polar interactions with the antigen. On the other side of the long HCDR3, the LCDR1 and LCDR3 loops extend towards the tip of HCDR3. This face of the paratope protrudes significantly by approximately 12 Å compared to the other side of the binding site. The open nature of the face made by the heavy chain CDRs suggests that it might be involved in interacting with a bulky and protruding component of the antigen, whereas the elongated properties of the second site suggests that it reaches into a specific site or cavity on the antigen. Altogether, the crystal structures of these antibodies illustrate that they are potentially capable of interacting with their gp120 antigen via two distinct recognition sites.

**Table 1:** X-ray crystallography statistics.

Crystal	PGT121	PGT122	PGT123
**Data Collection**	SSRL 11-1	SSRL 11-1	APS 23-ID
Wavelength, Å	0.979	0.979	1.033
Space group	P2_1_2_1_2_1_	C2	P2_1_
Unit cell	65.0, 65.7, 159.2	210.3, 42.0, 45.1	84.9, 80.3, 90.4
a, b, c (Å)			
α, β, γ (°)	90, 90, 90	90, 100.3, 90	90, 93.9, 90
Fab per ASU	1	1	2
Resolution ( Å)*	40-2.8 (2.9-2.8)	40-1.75 (1.85-1.75)	30-2.5 (2.6-2.5)
Completeness*	99.5 (99.9)	97.7 (97.2)	99.7 (99.9)
Redundancy*	7.2 (7.4)	3.3 (3.2)	3.7 (3.8)
No. total reflections	125,999	130,871	156,538
No. unique reflections	17,409 (1,692)	38,560 (5,819)	42,069 (4,183)
I/σ*	19.8 (3.5)	11.4 (2.4)	9.2 (2.3)
R_sym_ † ^,^ *	8.8 (48.4)	6.4 (43.0)	11.8 (51.0)
**Refinement statistics**			
Resolution (Å)	19.9 – 2.8	38.3 – 1.75	29.1 – 2.5
No. reflections total/R_free_	17,353/870	38,554/1,921	41,693/2,086
R_cryst_/R_free_ ‡ ^, ^ §	18.4/23.7	19.8/24.2	20.7/26.5
RMSD bond length (Å)	0.011	0.007	0.011
RMSD bond angles (°)	1.45	1.17	1.23
Protein atoms/solvent atoms	3454/13	3262/248	6646/165
Wilson B-value ( Å^2^)	59.7	28.1	56.6
Overall average B-value (Å^2^)	52.4	30.7	45.5
Average B-value protein (Å^2^)	52.4	30.3	45.6
Average B-value solvent (Å^2^)	38.7	34.8	42.2
Ramachandran Preferred %	95.4	96.3	95.0
Allowed %	99.8	99.5	99.5
PDB ID	4JY4	4JY5	4JY6

* Values in parentheses are for the highest resolution shell.

† R_sym_ = Σ|I-<I>|/Σ<I>, where I is the observed intensity, and <I> is the average intensity of multiple observations of symmetry related reflections.

‡ R = Σhkl∥Fobs|−|Fcalc∥/Σhkl|Fobs|.

§ R_free_ calculated from 5% of the reflections excluded from refinement.

### Sequence conservation

Despite possessing highly similar structures, antibodies of the PGT121 family vary significantly in their sequences. Sequence identity in the heavy chain variable region between the three antibodies ranges between 74 and 78%, whereas sequence identity in the light chain variable region is on the order of 70–83% (Fig. 1C). Mapping of identical and dissimilar residues on the crystal structures of these antibodies identifies putative paratope regions that may have been maintained and optimized for antigen recognition. Indeed, CDRs H1, H2 and H3, which form the open face, are particularly well conserved across PGT121, 122 and 123 (conserved face 1, Fig. 1B). Another region with high conservation is the junction of LCDR3 and the tip of HCDR3 on the elongated face (conserved face 2, Fig. 1B). As expected, the electrostatic potential in these conserved regions of the paratope is also maintained in the three antibodies (Fig. S1). The significant degree of residue conservation in these regions suggests they are important in mediating broad HIV-1 neutralization.

### Paratope mapping by extensive alanine-scanning mutagenesis

**Figure 2:**
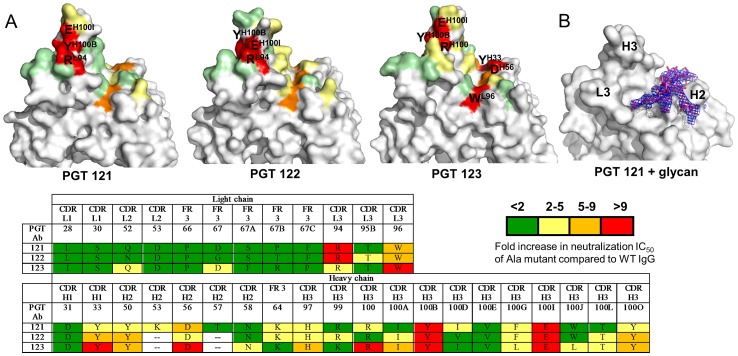
Alanine-scanning mutagenesis of the PGT121, PGT122 and PGT123 paratopes. A) Surface residues predicted to play a role in mediating antigen recognition were identified from the crystal structures, subsequently mutated to alanine, and the resulting IgG mutants were tested for their ability to neutralize HIV-1 JR-CSF pseudoviruses. The fold increase in the neutralization IC_50_ compared to WT IgG is reported in the table by color code: green, <2; yellow, 2–5; orange, 5–9; and red, >9. Mapping these results on the PGT121, PGT122 and PGT123 crystal structures allowed identification of a region crucial for mediating neutralization near the elongated face, whereas side chains of residues in the open face appear to play a more secondary role in HIV neutralization. Residues that show an increase in the neutralization IC_50_ of >9 (red) compared to WT IgG upon mutation to alanine are labeled on the structures. B) The PGT121 open face accommodates a biantennary glycan from a symmetry-related Fab molecule in the crystal (Figs. S2, S3). PGT121 is rendered as a gray surface, whereas the glycan is shown as magenta sticks. The blue mesh is the 2Fo-Fc electron density map countered at a 1.2 sigma level around the glycan moiety. The figure was generated using Pymol.

Based on the structure and sequence analysis described above, predicted key residues for antigen interaction were selected for all three antibodies in order to create alanine mutants for testing in HIV-1 pseudovirus neutralization assays. Mapping the effect of each mutation onto the crystal structures indicated that important paratope residues for HIV-1 neutralization map to highly conserved regions in the three antibodies (Fig. 2A). Two residues in the elongated conserved face 2 are critical for HIV-1 neutralization for all three antibodies: Tyr^H100B^ and Glu^H100I^. The drastic effect of mutating either one of these two residues to alanine in PGT121, PGT122 and PGT123 is suggestive that this conserved portion of the paratope is the primary determinant for antigen contact, and, hence, for HIV-1 neutralization. Other residues in this face, such as Arg^H100^ and Arg^L94^, also play crucial roles in mediating HIV-1 neutralization for individual antibodies. In the open face, the alanine-scanning mutagenesis has more moderate effects, but key residues important for HIV-1 neutralization in this region include HCDR1 Tyr^H33^, HCDR2 Tyr^H50^ and Asp^H56^, and LCDR3 Trp^L96^. We note that the PGT123 open face appears to play a more important role in mediating HIV-1 neutralization in these experiments, when compared to PGT121 and PGT122. Overall, the open face appears to represent a secondary site of interaction with the gp120 antigen.

### Glycan binding

The PGT121 family of antibodies is sensitive to an N332A mutation on gp120 for recognition [5]. The N332 glycan is reported to be predominantly of the unprocessed high-mannose type [23], [25], [26], [29]. As such, members of the PGT121 family were tested for their binding properties on a glycan-array containing several types of glycans, including high-mannose sugars. Surprisingly, the PGT121 family showed almost no reactivity to high-mannose sugars on the glycan array in contrast to bnAbs of the PGT128 family [5]. The lack of high affinity binding to high-mannose sugars was further validated by the absence of any electron density corresponding to glycans upon co-crystallizing PGT123 Fab with Man_9_GlcNAc_2_ or by soaking PGT123 Fab crystals in a high concentration solution of Man_9_GlcNAc_2_ prior to X-ray diffraction experiments (data not shown). On the other hand, PGT121 interaction with a complex biantennary glycan could be observed on the glycan array and in the crystal structure (Fig. 2B and Figs. S2, S3). Fortuitously, the glycan observed in the PGT121 paratope comes from an N-linked glycan of a crystal symmetry-related PGT121 Fab molecule, as Fab PGT121 is glycosylated and was expressed in mammalian cells (Fig. S1A). This glycan sits directly in the open face of the PGT121 paratope and buries 530 Å^2^ of antibody surface area [31]. Such binding of PGT121 to a complex glycan from a symmetry-related molecule has also been described in another crystal system ([30] and Fig. S2).

Alanine-mutagenesis experiments in the PGT121 paratope were performed to show that the residues that contact the complex glycan in the crystal structure are indeed responsible for binding complex sugars on the glycan array (Fig. S3). These data indicate that PGT121 Lys^H53^, which mediates two H-bonds to a galactose moiety of the biantennary complex glycan in the PGT121 crystal structure, confers complex glycan reactivity. Indeed, mutating this residue to an alanine completely abrogates binding to a complex glycan (Fig. S3D). In addition, PGT122 and PGT123 do not have a lysine at this position, but rather an Asp and a His, respectively, which might help to explain why these antibodies are not reactive with complex glycans on a glycan array. However, it appears that PGT121 reactivity with a complex sugar is not absolutely required for HIV-1 neutralization, since antibodies of the PGT121 family can neutralize viruses that were produced in the presence of kifunensine and, hence, lack complex sugars (Fig. S3B). This notion is consistent with the PGT121 interaction with the complex glycan in the crystal, as it has significant interactions with the core mannose residues that are a common feature of all N-linked glycans. Other interactions observed in the PGT121 crystal structure with specific glycan moieties of the complex biantennary might therefore not be essential for mediating HIV-1 neutralization.

### Binding to soluble recombinant Env trimers

**Figure 3:**
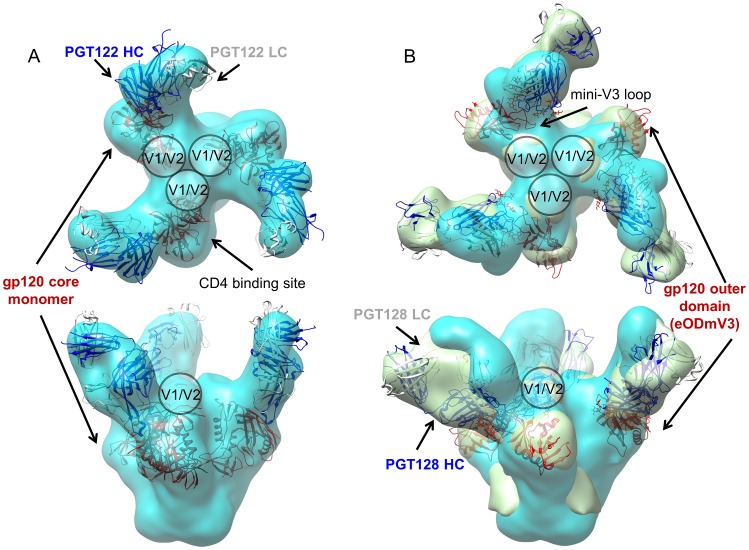
Negative stain electron microscopy reconstruction of HIV SOSIP.664 trimer in complex with Fab PGT122 and in comparison to PGT128 [**29**] **.** A) Top and side views of the BG505 SOSIP.664:PGT122 Fab reconstruction at 15 Å resolution with the fitted crystal structures of PGT122 Fab and gp120 core (PDB ID 3DDN [35]) rendered as secondary structure cartoons. The PGT122 Fab is shown in blue (heavy chain) and white (light chain), and the gp120 core is shown in red. B) Top and side views of the BG505 SOSIP.664:PGT122 Fab reconstruction at 15 Å resolution (cyan) and the KNH1144 SOSIP.664:PGT128 Fab reconstruction at 14 Å resolution [29] (light green), with the fitted crystal structure of PGT128:eODmV3 (PDB ID 3TYG [29]) shown as a secondary structure cartoon. The PGT128 Fab is shown in blue (heavy chain) and white (light chain), and the engineered gp120 outer domain (eODmV3) is shown in red. The proposed locations of V1/V2 loops and the V3 loop in the Env trimer, as well as the location of the CD4 binding site, have been labeled. The figure was generated using UCSF Chimera [70].

Despite isolating pure complexes of members of the PGT121 family with various gp120 constructs, we have not yet been able to obtain crystals that enable an atomic-level characterization of the epitope. Notwithstanding, negative-stain electron microscopy (EM) studies enabled characterization of the PGT122 epitope on the soluble HIV-1 SOSIP.664 gp140 trimer [32], [33] at 15 Å resolution. The EM reconstruction allowed unequivocal identification of three PGT122 Fab molecules bound per recombinant HIV-1 trimer and elucidation of the binding site and mode of interaction. As the dimple in the Fab created by the separation of the variable and constant domains is clearly visible in negative-stain EM, it was possible to accurately place and correctly orient the PGT122 Fabs on gp120 by also using restraints that come from knowledge of the Fab elbow angle (133.2° for PGT122 [34]) in the density fitting, as well as by using Protein G to identify and locate the C_H_1 domain of the Fab (Fig. S5). PGT122 interacts with the spike at an approximately 120° angle in relation to the viral membrane surface if the Env trimer threefold axis is aligned perpendicular to the membrane (Fig. 3A). The EM reconstruction clearly reveals that the PGT122 epitope resides on the gp120 outer domain opposite to the CD4 binding site, which is consistent with the previous report that the N332 glycan at the base of the V3 loop is a crucial component for PGT121 family recognition [5]. Comparison of the SOSIP.664:PGT122 Fab reconstruction with our previous SOSIP.664:PGT128 Fab reconstruction [29] allowed identification of key differences between the recognition of these N332-dependent epitopes by these two classes of antibodies isolated from different donors (Fig. 3B). Interestingly, the angle of approach to the recombinant Env trimer surface by these two antibodies is different. PGT128 Fab approaches at an angle slightly more parallel to the relatively flat apex of the recombinant Env trimer as opposed to a more vertical approach seen for PGT122 Fab. The PGT128 angle of approach leaves the apex exposed, where the V1/V2 loops are located [29]. On the other hand, PGT122 binding to the recombinant Env trimer partially masks elements of V1/V2 at the spike apex, which appears flat and resembles the closed conformation adopted by the unliganded trimer [35]. Steric clashes might therefore help explain the previously reported competition between PGT121 antibodies and PG9 [5].

### Allosteric modulation of gp120 conformation

**Figure 4:**
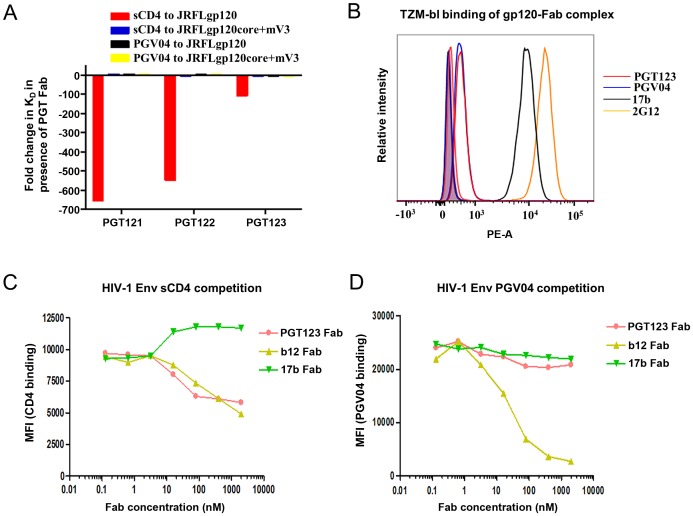
Antibodies of the PGT121 family compete with sCD4, but not PGV04, for binding to gp120 in solution and on the cell-surface. A. Fold change in binding affinity (K_d_) for sCD4 and PGV04 interactions with gp120 when antibodies of the PGT121 family are first pre-complexed with gp120. The order from left to right is as on the inset legend from top to bottom. Negative values indicate a decrease in binding affinity of sCD4 or PGV04 in the presence of PGT121 antibodies, positive values represent an increase in binding affinity and a value of ∼1 represents no effective change in binding affinity. Sequential ITC binding experiments show that sCD4 has over 100 to 650 fold decrease in binding affinity to gp120 when antibodies of the PGT121 family are pre-complexed with gp120. On the other hand, pre-complexing PGT121 antibodies with gp120 does not lead to a change in binding affinity for PGV04 to gp120. These results suggest that antibodies of the PGT121 family do not sterically block access to the CD4 binding site (Fig. S8A), but disturb CD4 binding, possibly by interfering with gp120 conformational changes associated with CD4 binding. No sCD4 competition by PGT121 antibodies is observed for a core-miniV3 gp120 construct, indicating that modulatory elements of gp120 such as C1, V1/V2 and fully length V3 are important in modulating sCD4 competition. B. SEC-purified complexes of gp120 with various Fabs were tested for their ability to bind CD4+ TZM-bl cells by FACS. 17b+gp120 (black) and 2G12+gp120 (orange) complexes bound well to CD4+ TZM-bl cells. On the other hand, a PGT123+gp120 complex (red) was not able to engage CD4+ TZM-bl cells. Lack of binding of the PGT123+gp120 complex to CD4+ TZM-bl cells is comparable to similar lack of binding of gp120 in complex with the CD4-binding site antibody PGV04 (blue). A similar inability to bind CD4+ TZM-bl cells was observed for gp120 in complex with PGT121 and PGT122 antibodies (Fig. S9). PE-A represents the relative intensity of detected Fab on the surface of CD4+ TZM-bl cells. Curves with filled areas indicate Fab alone (negative control), whereas curves with hollow areas are for the Fab-gp120 complexes. C) sCD4 and D) PGV04 binding to cells expressing JRFL Env on their surface as observed by flow cytometry. PGT123 Fab (pink), b12 Fab (yellow) and 17b Fab (green) were pre-incubated with the cells in titrating amounts at 37°C before being exposed to a constant amount of either C) sCD4 or D) PGV04. As expected, b12 Fab that targets the CD4 binding site directly competes with CD4 binding, and 17b Fab, which binds to the co-receptor binding site, enhances CD4 binding. PGT123 Fab competes with sCD4 binding to the same extent as b12 Fab. A similar level of sCD4 competition was observed for PGT121 and PGT122 antibodies (Fig. S9). On the other hand, PGT123 Fab does not compete with PGV04, a CD4 binding site targeted antibody that does not induce conformational changes upon binding [36]. Binding curves are represented by plotting the dimensionless mean fluorescence intensity (MFI) of C) sCD4 and D) PGV04 binding as a function of Fab concentration.

It is well established that interaction of CD4 with HIV-1 Env causes conformational changes in the trimer leading to a more open conformation that exposes the co-receptor binding site [35]. While it was previously noted that CD4 binding site antibodies do not induce such conformational changes in the trimer, some CD4 binding site antibodies do induce conformational changes in recombinant gp120 [36]. To probe any potential conformational changes induced in HIV-1 Env on binding the PGT 121 family, sequential binding experiments were performed. First, binding of antibodies of the PGT121 family to JRFL gp120 monomeric constructs was measured by isothermal titration calorimetry (ITC) (Figs. 4A and S6, S7) and resulted in binding affinities (K_d_) of 83–95 nM (Table 2). When soluble CD4 (sCD4) was then titrated into the monomeric gp120 solution saturated with antibodies of the PGT121 family, binding of sCD4 to monomeric gp120 significantly decreased from a K_d_ of 21 nM in the absence of PGT121 antibodies to a K_d_ of ∼10 µM in the presence of excess PGT121 antibodies (Figs. 4A and S6, S7 and Table 2). Together, these observations clearly indicate that antibodies of the PGT121 family compete with CD4 for binding to the gp120 monomer. In contrast, this competition was not observed for PGV04 Fab (Fig. 4A), which suggests that steric occlusion of the CD4 binding site is not the mechanism of competition utilized by antibodies of the PGT121 family. Interestingly, sCD4 competition was only observed in the context of a near full-length gp120 monomeric construct; deletions of elements of C1, V1/V2 and the V3 tip (gp120core+mV3) significantly decreased the binding affinity and did not result in sCD4 competition (Figs 4A and S6).

**Table 2:** Thermodynamic parameters of PGT 121 antibodies and CD4 binding to gp120 and SOSIP.664 trimers measured by isothermal titration calorimetry.

Binding experiment	ΔG% ^,^ #	ΔH%	−TΔS%	K_d_ %	N% ^,^ &
	(kcal mol^−1^)	(kcal mol^−1^)	(kcal mol^−1^)	(nM)	
sCD4 into gp120	−10.5	−40.0	29.5	21	0.7
PGT121 into gp120	−9.7	−31.4	21.7	86	0.5
PGT122 into gp120	−9.8	−40.0	30.2	95	0.5
PGT123 into gp120	−9.8	−37.1	27.3	83	0.5
sCD4 into (PGT121+gp120)	−6.6	—*	—*	13,400	—*
sCD4 into (PGT122+gp120)	−6.7	—*	—*	11,200	—*
sCD4 into (PGT123+gp120)	−7.8	—*	—*	2,200	—*
sCD4 into SOSIP.664	−8.5	−23.9	15.4	579	2.2
sCD4 into (PGT123+SOSIP.664)	minimal binding				
PGT123 into SOSIP.664	−8.2	−11.6	3.4	990	1.6
PGT123 into (sCD4+SOSIP.664)	minimal binding				

% Reported values are averages from at least two independent measurements and associated errors are approximately 10% of the average. Representative isotherms can be found in Figs. S6, S7.

* The binding isotherms do not allow to accurately determining these binding parameters.

# The change in Gibbs free energy (ΔG) was determined using the relationship: ΔG_binding_ = RTlnK_d_ [75].

& The stoichiometry of binding (N) is directly affected by errors in protein concentration measurements, sample impurity and glycan heterogeneity on gp120.

To confirm that such competition is also present on a recombinant HIV-1 trimer, the equivalent sequential ITC binding experiment was repeated with the soluble SOSIP.664 gp140 trimer. While sCD4 bound to the SOSIP.664 trimer with a K_d_ of 579 nM in the absence of PGT123 Fab, there was negligible binding in presence of PGT123 Fab (Fig. S7 and Table 2). PGT122 also competed with binding of the 337.8 Da CD4-like small molecule mimic NBD-556 in this system (Fig S8). Like sCD4, this molecule has been associated with inducing conformational changes in gp120 upon binding [37], [38], [39]. The reverse sequential binding experiment also revealed that PGT123 Fab was not able to bind SOSIP.664 trimer after pre-incubation with sCD4, despite binding the unliganded SOSIP.664 trimer with a K_d_ of 990 nM (Fig. S7 and Table 2). Therefore, the ITC experiments suggest the PGT121 antibody family must bind prior to receptor engagement by Env. Also noteworthy is the lower binding affinity of PGT123 Fab and sCD4 for the SOSIP.664 trimer, compared to the gp120 monomer (Table 2). One implication is that binding to the recombinant trimer is more constrained than to the monomer, as previously observed by others [40], [41], [42], [43]. However, we note that the gp120 that was used was from the JR-FL sequence, while the trimer was based on KNH1144. Additional studies with sequence-matched Env proteins, and possessing an identical glycosylation profile confirmed by mass spectrometry, will need to be performed to provide equivalent comparisons of binding to gp120 and soluble trimers.

Finally, to confirm the CD4-PGT121 competition in a more biologically relevant setting, fluorescence activated cell sorting (FACS) experiments were performed. First, gp120-Fab complexes were purified by size-exclusion chromatography and the complexes were subsequently tested for their ability to bind CD4+ TZM-bl cells. As expected from the solution binding experiments, gp120-PGT121 antibody complexes were not able to significantly bind to the CD4 receptor on TZM-bl cells, whereas gp120-17b (co-receptor binding site antibody) and gp120-2G12 (N332-dependent antibody) complexes did bind to cell surface CD4 (Figs. 4B and S9A). Moreover, the ability of JRFL Env-expressing cells to interact with CD4 was investigated after pre-incubation with different Fabs with known epitopes. Antibodies of the PGT121 family interfered with CD4 binding to Env at the cell surface to a similar extent as b12 Fab, a CD4 binding site antibody (Fig. 4C and S9C). We note, however, that surprisingly high concentrations of Fabs were required to achieve CD4 competition, which did not reach 100% binding saturation (Figs. 4C and S9C). Whether this is specific to the assay or whether it is intrinsic to the mechanism of competition by these Fabs remains to be determined. On the other hand, 17b Fab binding increased the ability of Env-expressing cells to interact with CD4 in this assay (Fig. 4C), most likely due to favorable conformational changes induced in Env upon recognition by this co-receptor binding site antibody [44]. Steric occlusion was again ruled out as a competition mechanism since antibodies of the PGT121 family did not compete with the CD4 binding site bnAbs PGV04 and VRC01 for binding to cell-surface trimers (Figs. 4C and S9D). The FACS data, therefore, corroborate the ITC binding and TZM-bl binding results that illustrate how antibodies of the PGT121 family can compete with and prevent CD4 binding after their interaction with gp120. Because the PGT121 family epitope is significantly distant from the CD4 binding site as observed by electron microscopy (Fig. S8), a likely explanation for the observed inhibition of CD4 binding by antibodies of the PGT121 family is allosteric blockade of conformational rearrangements that are required for the gp120 components to adopt a conformation optimal for CD4 binding.

## Discussion

**Figure 5:**
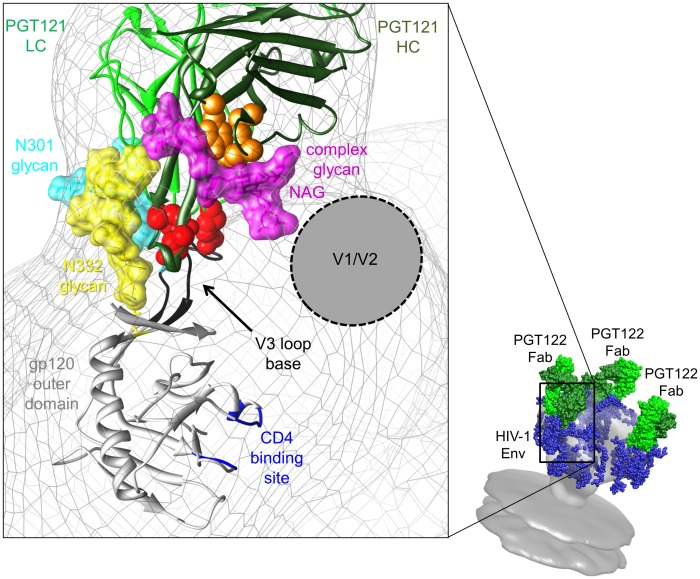
Model of HIV Env recognition by antibodies of the PGT121 family. A model of the PGT 121 family interaction with Env trimer is shown on the bottom right and was generated from the electron microscopy reconstruction of the unliganded membrane-anchored HIV-1 Env trimer (gray surface, EMDB ID 5019 and 5021 [35]) with modeled glycans and PGT121 Fab as blue spheres and green surface, respectively. The large inset is a close-up view of the fitting of the PGT121 Fab crystal structure (light and heavy chains are colored in light and dark green, respectively) and the eODmV3 crystal structure (colored in gray), as bound by the PGT128 Fab (PDB ID 3TYG [29]) in the negative-stain EM reconstruction (transparent gray mesh). The crystal structures of the PGT121 Fab and the gp120 outer domain are rendered as secondary structure cartoons. PGT121 paratope residues identified as most important for mediating HIV-1 neutralization are shown as red and orange spheres according to the scheme used in Fig. 2. In this model, crucial residues of the PGT121 paratope in the elongated face (rendered as red spheres) are located near the base of the gp120 V3 loop (black). Consistent with the biochemical data, the antibody paratope is located near the N332 glycan (rendered as yellow sticks and surface). Glycosylation at this position is crucial for recognition by antibodies of the PGT121 family. In addition, the PGT121 paratope is located in close proximity to the N301 glycan (rendered as cyan sticks and surface), as well as putative complex glycan (rendered as magenta sticks and surface) observed in the PGT121 crystal structure of unknown location on gp120 but close to V1/V2 in the model. For this complex glycan, the two N-acetylglucosamines (NAG) that would be attached to the Asn on the protein point in the direction of the region associated with gp120 V1/V2 loops. Together, our data show that PGT 121 binding to this epitope appears to allosterically block CD4 engagement. The CD4 binding site is colored blue. The figure was generated using UCSF Chimera [70].

One key strategy guiding HIV-1 vaccine design has been the identification of sites of vulnerability on the Env protein via the characterization of epitopes recognized by broad and potent neutralizing antibodies. Here, we extensively characterize a new class of bnAbs, the PGT121 family, which was previously shown to neutralize approximately 70% of circulating HIV-1 isolates and recognizes an N332-dependent epitope on the gp120 outer domain [5]. Structural characterization of PGT122 Fab in complex with a recombinant HIV-1 Env trimer by electron microscopy reveals the location of the epitope and a novel angle of approach to the N332-dependent site of vulnerability on the gp120 outer domain for this family of antibodies. A combined approach of neutralization assays with antibody point mutants, and the fitting of high resolution crystal structures of individual components in the EM reconstruction, allow us to propose that the primary site for antibody recognition consists of the N332 glycan, and possibly another glycan, as well as protein-protein interactions near the base of the V3 loop (Fig. 5). Accordingly, previous alanine-scanning mutagenesis studies on gp120 revealed that, other than the glycosylation site at position N332 that was essential for Env recognition, other mutations that had moderate effects on JR-CSF neutralization by this class of antibodies were Asp325 and Ile326 (base of the V3 loop) [5]. In our proposed model, these residues at the base of the V3 loop are directly adjacent to critical paratope residues of the elongated face identified by alanine-scanning mutagenesis: Arg^L94^, Tyr^H100B^ and Glu^H100I^. We propose that these residues form the primary and most critical site of epitope-paratope interactions. Other possible sites of interaction between the antibody and gp120 based on the EM model include gp120 strands β19 and β22. These strands are in close proximity to the V3 loop and lead into and emanate from the β20 and β21 strands that take part in forming the bridging sheet. In accordance with this hypothesis, Ile420 and Ile423 (β19/β20 strands) were also previously shown to have moderate effects on JR-CSF neutralization by antibodies of the PGT121 family [5].

As revealed by the lack of binding on the glycan array, the affinity of antibodies of the PGT121 family for isolated high mannose glycans is relatively weak compared to other glycan-dependent anti-HIV-1 antibodies, such as bnAbs of the PGT128 family and 2G12 [29]. This apparent lack of binding on the array does not preclude the possibility that PGT121-like antibodies interact more strongly with glycans in the context of gp120, especially those surrounding the gp120 V3 base. As previously observed in the PGT128-gp120 outer domain crystal structure, as well as in the EM characterization of the PGT128 interaction with a recombinant SOSIP.664 gp140 trimer, the base of the V3 loop harbors two critical glycans at positions N301 and N332 [29]. Combining these data with our EM characterization of the PGT122-SOSIP.664 gp140 trimer interaction suggests putative interactions between antibodies of the PGT121 family and the N301 and N332 glycans. Our model predicts that the N301 and N332 glycans might be accommodated by elements of the light chain CDRs and at the interface between light chain CDRs and HCDR3, respectively (Fig. 5). This model is particularly attractive because it places the high mannose N332 glycan, on which HIV-1 neutralization by the PGT121 family relies heavily, as a central component of the epitope. Harder to interpret in the context of HIV-1 Env recognition, however, is the ability of PGT121 to interact with a complex biantennary glycan in glycan arrays and in crystal structures [30]. Modeling of the “PGT121-liganded” crystal structure in our EM density reveals that the complex glycan is adjacent to the N332 glycan. In this orientation in the model, the first N-acetyl-glucosamine (NAG) of the glycan emanates from density attributable to elements of gp120 V1/V2, for which little structural information is known in the context of gp120. Our model therefore suggests that the open face of the PGT121 antibody could possibly accommodate another glycan on gp120 in addition to the N332 glycan, potentially from the gp120 V1/V2 loops. Overall, the mode of gp120 protein-glycan recognition by the PGT121 antibody family might, therefore, in some ways resemble PG9 recognition of the gp120 V1/V2 domain. In this system, the elongated HCDR3 in PG9 penetrates the glycan coat to access a conserved polypeptide epitope comprising a cationic grove on gp120 V1/V2 that is juxtaposed to its surrounding glycans, facilitating paratope interactions with the glycans which enhance binding affinity [45]. Notably, only millimolar affinity was detected for PG9 carbohydrate binding alone [45].

Because the gp120 CD4 binding site is on the opposite face of gp120 relative to the PGT122 epitope, the mechanism by which it effectively neutralizes HIV-1 infection remains unclear [29], [46]. Surprisingly, binding of PGT121 antibodies to their epitope interferes CD4 binding to gp120. The disruption of CD4 binding by PGT121 antibodies is observed in the context of recombinant monomeric gp120, recombinant trimeric gp140 and cell-surface Env trimers. Lack of competition with PGV04 and VRC01, but the presence of competition with CD4, is an argument against steric effects of PGT121 being the main cause of interference with CD4 binding to gp120. Combined with a relatively closed structure for the PGT122-SOSIP.664 trimer complex, these data lead us to suggest that the PGT121 antibody family effects HIV-1 neutralization by locking functional Env molecules in a conformation that prevents productive CD4 receptor engagement. However, simply binding to the high mannose patch bearing the N332-glycan might not be sufficient to confer such allosteric modulation of the CD4 binding site. Indeed, we show that in contrast to the PGT121 family, 2G12 fails to significantly prevent gp120 from binding CD4+ TZM-bl cells. These results agree with previous reports that show that 2G12 inhibits cell entry predominantly via competition with CCR5, although the effect of this antibody on CD4 binding to gp120 appears to vary in different assay systems [46], [47]. A recent report investigating the structure of unliganded gp120 monomers has suggested that C1 and the variable regions V1/V2 and V3 in gp120 might have profound effect in modulating the conformational changes associated with CD4 engagement [39]. Taken together with our observations, we suggest that antibodies, such as the PGT121 family, that involve protein elements of the V3 base in their epitope in addition to surrounding glycans, and perhaps with involvement of V1/V2, might constrain the Env trimer spikes and, hence, allosterically inhibit receptor binding, co-receptor binding and membrane fusion. We note that, in the cell-surface competition assays, the concentration at which CD4 competition is achieved appears higher than that required to achieve HIV-1 neutralization. Therefore, the physiological relevance to neutralization remains to be confirmed and other neutralization mechanisms might also be in play for this class of antibodies, such as induction of viral decay, for example [29]. Future studies will determine whether restraining the HIV-1 spike in a closed conformation is a major mechanism of neutralization and whether other bnAb families are also capable of preventing CD4 binding through an allosteric mechanism.

## Materials and Methods

### Protein expression, purification, crystallization and X-ray diffraction

JRFL gp120 monomeric constructs were expressed and purified as previously described [29]. Briefly, the gp120 genes cloned adjacent to an IgK secretion signal in a phCMV3 plasmid were transfected in either HEK 293F cells or HEK 293S cells (GnT I-deficient) using 293Fectin (Invitrogen). The secreted gp120 monomer was recovered 6-days post-transfection and purified by GNL affinity chromatography followed by gel filtration chromatography. A detailed protocol for the construction, expression and purification of the SOSIP.664 construct of Clade A KNH1144 sequence in HEK 293S GnT I-deficient cells can be found elsewhere [32], [33]. Briefly, following expression, the secreted SOSIP.664 construct was harvested from the supernatant and purified using a 2G12-coupled affinity matrix. SOSIP.664 of Clade A BG505 sequence with a T332N point mutation was used in EM experiments and was obtained using an identical protocol. sCD4 was expressed in bacteria as inclusion bodies, refolded and purified via nickel affinity using a protocol similar to that previously described [48].

PGT121 and 122 Fab were produced in 293T cells and secreted in the expression media as previously described [49]. To create the Fab fragment, the heavy chain IgG gene was first mutated by m-PIPE cloning to introduce a stop codon directly after the cysteine involved in the Fab heterodimer disulfide [50]. To obtain crystals with better diffracting properties, it was necessary to remove the glycosylation site on the PGT122 Fab by site-directed mutagenesis introducing an N to Q mutation. Subsequently, the heavy and light chain genes were co-transfected into HEK 293T cells. Three days after transfection, the expression media was harvested and purified via an anti-human λ light chain affinity matrix (CaptureSelect Fab λ; BAC), followed by cation exchange chromatography and size-exclusion chromatography. PGT123 Fab was produced in Sf9 insect cells via baculovirus infection and secreted in the expression media as previously described [51]. Initially, a pFastBacDual (Invitrogen) plasmid was created that contained both the Fab heavy chain and light chain genes preceded by a gp67 secretion signal. Production of the bacmid and recombinant baculovirus was performed using the manufacturer's Bac-to-Bac TOPO Expression System protocol (Invitrogen). The supernatant of infected Sf9 cells was harvested 3 days after infection and the purification method was identical to that of the Fabs produced in mammalian cells. After purification, Fab fragments were concentrated to ∼10 mg/mL and setup for crystallization trials using the automated CrystalMation robotic system (Rigaku) at the Joint Center for Structural Genomics (www.jcsg.org). X-ray diffraction quality crystals were obtained in the following conditions: PGT121 Fab: 0.1 M Hepes, pH 7.5, 30% v/v PEG 400, 5% w/v PEG 3000, 10% v/v glycerol; PGT122 Fab: 0.1 M CAPS, pH 10.5, 0.2 M sodium chloride, 20% w/v PEG 8000; PGT123 Fab: 0.16 M zinc acetate, 0.08 M sodium cacodylate, pH 6.5, 20% v/v glycerol, 20% w/v PEG 8K. For PGT122 Fab, the mother liquor was supplemented with 20% glycerol for cryo-protection. Full datasets for the PGT121, PGT122 and PGT123 crystals were collected at the SSRL 11-1 and APS 23-ID beamlines. Data processing was performed using XDS [52]. The structure for PGT123 Fab was solved in space group P2_1_ using Fab coordinates PDB ID 3FN0 as a search model in PHASER [53]. Subsequently, the PGT121 and PGT122 structures were solved also using PHASER in space groups P2_1_2_1_2_1_ and C2, respectively with the PGT123 Fab structure as a search model. Refinement of the structures was performed using a combination of CNS [54], CCP4 [55], PHENIX [56] and COOT [57]. The final statistics for all Fab structures are reported in Table 1.

### Paratope mutants and neutralization assays

Mutations were introduced using QuikChange site-directed mutagenesis (Stratagene) following the manufacturer's protocol. Mutants were verified by DNA sequencing. Pseudoviruses were generated by transfection of 293T cells with a JR-CSF HIV-1 Env expressing plasmid and an Env-deficient genomic backbone plasmid (pSG3ΔEnv), as described previously [58]. Kifunensine-treated pseudoviruses were prepared by addition of kifunensine (final concentration of 25 µM) at the time of transfection [59]. Pseudoviruses were harvested 72 h post-transfection for use in neutralization assays. Neutralizing activity was assessed using a single round of replication pseudovirus assay and TZM-bl target cells, as described previously [58]. Briefly, TZM-bl cells were seeded in a 96-well flat bottom plate. To this plate was added pseudovirus, which was preincubated with serial dilutions of antibody for 1 h at 37°C. Luciferase reporter gene expression was quantified 72 h after infection upon lysis and addition of Bright-Glo Luciferase substrate (Promega). To determine IC_50_ values, dose–response curves were fitted using nonlinear regression.

### Glycan array

PGTs 121–123 IgGs were screened on a printed glycan microarray version 5.0 from the Consortium for Functional Glycomics (CFG) as described previously [60]. Amine-functionalized sugars were printed in replicates of six onto NHS-activated glass slides at a concentration of 100 µM using a MicroGridII contact microarray printing robot [61], [62]. Antibodies (30 µg/mL in 3% BSA and 0.05% Tween-20 in PBS) were pre-complexed with goat-anti-human-Fcγ-R-PE (15 µg/mL, Jackson) for 10 min at room temperature. The sample was added to the glycan array and incubated at room temperature for 1 h. The slides were washed sequentially in PBS/0.05% Tween-20, PBS and water. Arrays were scanned for R-PE fluorescence on a ProScanArray HT (PerkinElmer) confocal slide scanner at 70PMT90LP. Signal intensities were collected using Imagene (BioDiscovery) image analysis software and calculated using the mean intensity of 4 replicate spotted samples. Complete glycan array data sets for these antibodies can be found at www.functionalglycomics.org in the CFG data archive under “cfg_rRequest_2250”.

### Electron microscopy

Negatively stained grids were prepared by applying 0.1 mg/mL of the purified SOSIP.664 in complex with PGT122 Fab to a freshly glow discharged carbon coated 400 Cu mesh grid and stained with 2% Nano-W (Nanoprobes). Grids were viewed using a FEI Tecnai TF20 electron microscope operating at 120 kV and imaged at a magnification of 100,000×. Images were acquired on a Gatan 4 k×4 k CCD camera in five degree increments from 0–55° tilt angles at a defocus range of 600 to 720 nm and less than 16 e-/Å^−2^ using LEGINON [63]. The tilt angles provided additional particle orientations to improve the image reconstructions. The pixel size of the CCD camera was calibrated at this magnification to be 1.09 Å using a 2D catalase crystal with known cell parameters.

A cross-linked PGT122Fab∶ProteinG∶SOSIP.664 sample was prepared by incubating the components in a 6∶10∶1 molar ratio, adding 0.01% gluteraldehyde and subsequently purifying the resulting mixture to homogeneity on a Superose 6 size-exclusion column. Grids of this sample were prepared as indicated above. Data were collected using a Tecnai T12 electron microscope operating at 120 kV at 67,000× magnification using a dose of 25 e-/Å^−2^. Images were acquired on a Tietz 2 k×2 k CCD camera using LEGINON [63] at a defocus range of 500 to 1000 nm. The pixel size of the CCD was determined to be 2.05 Å at this magnification.

### Data collection and image reconstruction

All particles were automatically selected from micrographs with DoG Picker [64]. Contrast Transfer function (CTF) estimation for the untilted and tilted micrographs was determined with ctffind3 and ctftilt [65]. Particles were binned by 4 (80×80 sized boxes) and reference free 2D class averages were calculated using the Sparx package (Fig. S4) [66]. Forty *ab initio* models were generated from the final reference-free 2D class averages using the EMAN2 package. Each model was then refined against the reference-free 2D class averages using Sparx [66], [67]. The model exhibiting Fab-like density was used as the initial model for iterative image reconstruction against the CTF corrected particles using Sparx [66]. The resolution of the final image reconstruction, as determined by a Fourier shell correlation (FSC) of 0.5 is 15 Å (Fig. S4). The Protein G cross-linked particles were boxed out without CTF correction. Particles were binned by 2 (80×80 sized boxes) and reference free class averages were calculated using Sparx [66]. Particles with bound Fabs were selected into a substack, and re-filtered for classes with Protein G density based on reference free 2D class averages generated by Xmipp Clustering and 2D alignment [68]. The *ab initio* model from above was used for refinement against the raw particle stack of the Protein G bound complex, using Sparx [66]. The resolution of the final model was determined to be 22 Å using an FSC cut-off of 0.5.

### Enantiomer detection of image reconstruction and model fitting

The correct enantiomer of the image reconstruction was determined by fitting the crystal structures of the PGT122 Fab and the gp120 core (PDB ID 3DNN [35]) into each enantiomer using the program Molrep with C3 symmetry [69]. The PGT122 Fab and gp120 crystal structures fit the same enantiomer with a higher correlation coefficient than the opposite enantiomer (Table S1). Subsequently, the orientation of the Fab, with respect to its long axis, was further interrogated using the “fit” command of UCSF Chimera [70]. The fit with highest correlation in Molrep positioned the PGT122 with its CDR loops within the density and interacting with the gp120 structure (Fig. S5). This docking was further confirmed by the Protein G-bound EM structure.

### Isothermal Titration Calorimetry

Binding experiments were performed by isothermal titration calorimetry using either a MicroCal iTC200 or an Auto-iTC 200 instrument (GE Healthcare). Before conducting the titrations, all proteins were extensively dialyzed against a buffer consisting of 20 mM Tris, 150 mM NaCl, pH 8.0. Protein concentrations were subsequently determined and adjusted as required by absorbance at 280 nm using calculated extinction coefficients [71]. The ligand present in the syringe was either PGT121 Fab, PGT122 Fab, PGT123 Fab, PGV04 Fab or sCD4 at concentrations ranging between 57 µM and 121 µM. The gp120 monomer or SOSIP.664 trimer were in the cell at concentrations ranging between 6.3 µM and 9.9 µM. One experiment consisted of 16 injections of 2.5 µL each, with injection duration of 5 s, injection interval of 180 s and reference power of 5 µcals. To perform sequential binding experiments by ITC, the mixed sample from the first titration was left in the cell and the concentration of the HIV-1 component was recalculated based on the dilution from the first experiment (approximately ∼88% of the initial concentration). Subsequently, either Fab or sCD4 was added in a second titration. Origin 7.0 software was used to derive the affinity constants (K_d_), the molar reaction enthalpy (ΔH) and the stoichiometry of binding (N) by fitting the integrated titration peaks using a single-site binding model. From the change in Gibbs free energy, ΔG, the entropic change ΔS could also be calculated. All measured and derived thermodynamic parameters of binding are reported in Table 2.

### CD4+ TZM-bl cells binding assays

PGT121 Fab, PGT122 Fab, PGT123 Fab, PGV04 Fab, 2G12 (Fab)_2_′ and 17b Fab were mixed in excess with gp120. Complexes were purified by size-exclusion chromatography using a Superdex 200 16/60 column. Confluent TZM-bl cells were harvested using 10 mM EDTA (Invitrogen) in PBS. The purified complexes were then added to the TZM-bl cells to a final constant concentration of 100 µg/mL. To ensure the equilibrium was shifted to favor complex formation, the solution was supplemented with corresponding Fabs to a final concentration of 200 µg/mL. After incubation for 1 h at room temperature, the cells were then washed twice with PBS and stained with a 1∶200 dilution of anti-F(ab)2 -R-phycoerythrin (BD Biosciences) for 1 h at room temperature. The cells were then washed twice and binding was measured by flow cytometry (BD LSR II Flow Cytometer) and analyzed using FlowJo software. Binding was represented by histograms.

### Cell surface binding assays

Titrating amounts of PGT121 Fab, PGT122 Fab, PGT123 Fab, b12 Fab and 17b Fab starting at 100 µg/mL and diluted 5-fold were added to JR-FLΔCT Env transfected 293T cells and incubated for 30 min at 37°C. After the initial incubation, constant amounts of either sCD4 at 5 µg/mL, biotinylated-PGV04 at 5 µg/mL or biotinylated-VRC01 at 5 µg/mL were added to each well containing the Fabs and incubated for 1 h at 37°C. Cells were then washed 2× with FACS buffer and stained with either a 1∶5 dilution of anti-CD4 v4 antibody conjugated to R-PE (BD biosciences) or R-PE-conjugated F(ab′)_2_ goat anti-human IgG specific for the Fc fragment (Jackson ImmunoResearch) at a 1∶200 dilution. Binding was analyzed using flow cytometry, and binding curves were generated by plotting the mean fluorescence intensity of sCD4, PGV04 or VRC01 binding as a function of Fab concentration. A FACSArray plate reader (BD Biosciences) was used for flow cytometric analysis and FlowJo software was used for data interpretation.

### Data deposition

Coordinates and structure factors for PGT121, PGT122 and PGT123 Fab structures have been deposited with the Protein Data Bank accession codes: 4JY4, 4JY5 and 4JY6. The SOSIP.664:PGT122 Fab EM reconstruction density has been deposited with the Electron Microscopy Data Bank accession code EMD-5624.

## Supporting Information

Figure S1 **Electrostatic rendering of the paratope of PGT121 antibodies.** Surfaces are colored according to their electrostatic potential, with red, blue and white surfaces representing regions of negative, positive and neutral electrostatic potential, respectively. Overall, the elongated and open faces of the PGT121 antibodies have similar electrostatic potential properties, particularly in the conserved regions detailed in Fig. 1B. Only the antibody F_v_ region is shown for clarity. This figure was generated using UCSF Chimera [70].
(TIF)

Figure S2 **The crystal packing of the PGT121 antibody structure results in a complex glycan located in the antibody paratope.** A) Assembly of PGT121 Fab in the crystal lattice in space group P2_1_2_1_2_1_ with unit cell dimensions of a = 65.0 Å, b = 65.7 Å, c = 159.2 Å (magenta box). Calculations of the Matthews' coefficient indicate a V_m_ = 3.59 Å^3^/Da and 65.8% solvent content with one molecule per asymmetric unit [73], [74]. The PGT121 Fab is rendered as secondary structure cartoon with symmetry-related molecules colored differently. The glycan on PGT121 heavy chain framework 4 (HFR-4) is shown as spheres. B. Assembly of PGT121 Fab in the crystal lattice in space group P2_1_2_1_2_1_ with unit cell dimensions of a = 67.8 Å, b = 67.8 Å, c = 94.1 Å (magenta box), PDB ID 4FQC [30]. Calculations of the Matthews coefficient indicate a V_m_ = 2.29 Å^3^/Da and 46.2% solvent content with one molecule per asymmetric unit [73], [74]. The PGT121 Fab is rendered as secondary structure cartoon with symmetry-related molecules colored differently. The glycan on PGT121 HFR-4 is shown as spheres. Although the crystal packing in this system is significantly different than in A), it leads to the glycan moiety sitting in the same paratope region in both structures. C) As in PDB ID 4FQC, the crystal packing in the PGT121 structure reported here puts the symmetry-related biantennary carbohydrate from the heavy chain FR-4 in the paratope open face. This fortuitous interaction in the crystal allows an appreciation for understanding how the PGT121 paratope can accommodate glycan moieties in its paratope. D) Another view from that shown in Fig. 2B of the PGT121 open face binding to a biantennary glycan from a symmetry-related molecule in the crystal lattice. Rendering of PGT121 and the glycan is the same as in Fig. 2B. The blue mesh is a 2Fo-Fc electron density map contoured at a 1.2 sigma level around the glycan moiety. Here again, the two N-acetylglucosamines (NAG) that would be attached to the Asn on the protein are on the left and the mannose core on the right form the key interactions with PGT121. The figure was generated using Pymol.
(TIF)

Figure S3 **Glycan binding properties of antibodies of the PGT121 family.** A) In contrast to antibody 2G12, antibodies of the PGT121 family do not possess high affinity for glycan moieties representative of oligomannose motifs. PGT121 does, however, interact favorably with biantennary N-linked glycans terminating with one or two α-2–6 linked sialic acids. Thus, from these data, it is not known from which site on the Env trimer this glycan would emanate. Blue squares = N-acetyl glucosamine; green circles = mannose; yellow circles = galactose; pink diamond = sialic acid. RFU = relative fluorescence units. B) Although PGT121 is able to bind a complex sugar on the glycan array, it is able to neutralize JRFL pseudovirus made in the presence of kifunensine with a similar potency. Kifunensine-treated pseudovirus displays mainly Man_9_GlcNAc_2_ glycans and suggests that PGT121 reactivity with a biantennary complex sugar is not required for HIV-1 neutralization. C) Top view of the PGT121 paratope, color-coded by the importance of paratope residues on HIV-1 neutralization, as in Fig. 2. The observed biantennary glycan sits in the secondary “open-face” paratope groove, and hydrogen bonds are mediated by side-chain and backbone atoms of residues D^H31^, Y^H33^, K^H53^, G^H55^, N^H58^, R^H94^, H^H97^ and R^H99^. Of the hydrogen-bonding residues, only Y^H33^, K^H53^ and H^H97^ were identified by alanine-scanning mutagenesis as being moderately important in mediating HIV-1 neutralization. D) Alanine mutants of PGT121 paratope residues forming the glycan binding pocket knock out glycan binding, confirming that the glycan observed in the crystal structure is representative of binding to biantennary N-linked glycans terminated in 2–6 linked sialic acids in the glycan array. Particularly, the K^H53^A mutation abrogates binding to all glycan types. Lysine at position K^H53^ is only present in PGT121, and not in PGT122 and PGT123, possibly helping to explain the higher reactivity of PGT121 on the glycan array when compared to PGT122 and PGT123.
(TIF)

Figure S4 **Negative stain electron microscopy of the SOSIP.664:PGT122 Fab complex.** A) Reference free class averages of the SOSIP.664:PGT122 Fab complex calculated from 60 particle orientations are shown on the far left. Representative raw particles for each class average, low pass filtered to 15 Å resolution are shown at the right. The white bar in the first box on the upper left corresponds to ∼100 Å. B. Fourier shell correlation (FSC) curve of the SOSIP.664:PGT122 Fab complex image reconstruction. The curve measures the correlation between two independent image reconstructions as calculated from two halves of the entire data set (10,413 particles). The resolution of the image reconstruction is measured to be ∼15 Å resolution based on an FSC of 0.5.
(TIF)

Figure S5 **Fitting of the PGT122 Fab in the EM reconstruction of the SOSIP.664:PGT122 Fab+Protein G complex.** To ascertain the correct fitting of the PGT122 Fab from the two possible orientations in the EM reconstruction, Protein G was added to correctly position the C_H_1 fragment. The PGT122 Fab crystal structure (light and heavy chains are colored in light and dark green, respectively) and the eODmV3 crystal structure (PDB ID 3TYG [29]) (colored in gray) were fitted using the “Fit in map” tool in the negative-stain EM reconstruction (transparent gray mesh) using UCSF Chimera [70]. The Protein G - PGT122 Fab interaction was modeled according to the previously solved crystal structure of a mouse Fab in complex with Protein G (PDB ID 1IGC). The crystal structures are rendered as secondary structure cartoons. The model presented in A) most likely represents the correct orientation of PGT122 Fab binding to HIV-1 Env gp140 because of 1) an excellent fit of the Protein G model in the density; 2) an excellent fit of the F_v_ region; and 3) residues identified by alanine scanning mutagenesis as critical for mediating HIV-1 neutralization (red spheres) fall inside the EM density and point toward gp120 elements. These key requirements are not met in the alternate model presented in B). The higher correlation coefficient for model A) is indicative of the attributes mentioned above.
(TIF)

Figure S6 **Binding of PGT121 antibodies to different gp120 constructs, as evaluated in ITC experiments.** A) Bar graph showing the binding affinity (K_d_) of PGT121 antibodies for three different gp120 monomeric constructs. Binding of sCD4 was used as a control. A maximum binding affinity of ∼80 nM is observed for all three PGT121 antibodies. Expression in a cell-line leading to immature oligomannose glycans (HEK 293S cells) reduces the affinity moderately, whereas deletion of C1, V1/V2 and V3 tip results in a 10–100 fold decrease in binding affinity. B) Representative ITC binding isotherms for the data presented in A). The top panel shows representative raw data and the bottom panel is the binding isotherm. C) Sequence alignment of the two gp120 monomeric constructs used in the current study.
(TIF)

Figure S7 **Competition between PGT123 and sCD4 for binding to monomeric gp120 and SOSIP.664 gp140 trimers, as evaluated in ITC experiments.** The data are representative of those obtained with PGT121 and PGT122 antibodies. Top panel shows representative raw data and the bottom panel is the binding isotherm. A) ITC experiments of PGT123 Fab and sCD4 binding to a gp120 monomeric construct. Whereas individual binding experiments reveal high affinity binding (left panel and center panel), pre-incubation of monomeric gp120 with PGT123 leads to a significant loss in binding of sCD4, as observed from a decrease in both binding affinity and stoichiometry (right panel). B) Binding isotherms for mixing of sCD4 into SOSIP.664 (second panel) and into a pre-formed PGT123 Fab:SOSIP.664 complex (third panel). Although sCD4 binds well to the unliganded SOSIP trimer, pre-incubation of the SOSIP trimer with PGT123 Fab almost completely abrogates binding by sCD4, as evidenced by the lack of significant heat produced upon mixing. PGT123 Fab mixing with SOSIP.664 in ITC experiments results in saturating binding (first panel); however, pre-incubation of SOSIP.664 with sCD4 almost completely inhibits PGT123 Fab binding (fourth panel), suggesting that optimal PGT123 interaction with the HIV-1 Env trimer probably occurs prior to CD4 receptor engagement. Binding parameters for all ITC experiments are reported in Table 2.
(TIF)

Figure S8 **Competition between PGT122 and a small molecule sCD4 mimic, NBD-556, for binding to SOSIP.664 gp140 trimers, as evaluated in ITC experiments.** A) Model of PGT122 Fab interacting with the SOSIP.664 gp140 trimer. Rendering is as in Figs. 5 and S5. PGT122 Fab binding does not sterically occlude the CD4 binding site. Thus, the CD4 binding site should remain accessible for binding by sCD4 (red secondary structure cartoon) and a small molecular CD4 mimic of 337.8 Da, NBD-556 (orange sticks and surface). B) Binding isotherms for mixing of NBD-556 into SOSIP.664 (first panel) and into a pre-formed PGT122 Fab:SOSIP.664 complex (second panel). Although NBD-556 binds well to the unliganded SOSIP trimer (K_d_ = 1.7 µM), pre-incubation of the SOSIP trimer with PGT122 Fab almost completely abrogates binding by NBD-556, as evidenced by the lack of significant heat produced upon mixing. The top panel shows representative raw data and the bottom panel is the binding isotherm. Together, these data support the hypothesis that antibodies of the PGT121 family block interactions with CD4 binding site elements that induce conformational changes, such as sCD4 and NBD-556, by mechanism other than steric occlusion, and possibly through allostery.
(TIF)

Figure S9 **Competition between antibodies of the PGT121 family and CD4 for binding to cell surface components, as determined in FACS experiments.** A) SEC-purified complexes of gp120 with various Fabs were tested for their ability to bind CD4+ TZM-bl cells by FACS. Whereas 17b+gp120 (black) and 2G12+gp120 (orange) complexes bound well to CD4+ TZM-bl cells, PGT121+gp120, PGT122+gp120 and PGT123+gp120 complexes (red) were not able to engage CD4+ TZM-bl cells. Lack of binding of the gp120 complexes with PGT121, PGT122 and PGT123 is comparable to that of the CD4-binding site antibody PGV04 in complex with gp120 (blue). On the x-axis, PE-A represents the relative intensity of detected Fab on the surface of CD4+ TZM-bl cells. Filled areas indicate Fab alone (negative control), whereas hollow areas are for the Fab-gp120 complexes. B) Binding curves of elements used in the competition assays to cell-surface HIV-1 JRFL Env. C) sCD4 binding to cells expressing JRFL Env on their surface as observed by flow cytometry. PGT121 (brown), 122 (green) and 123 (blue) Fab were pre-incubated with the cells in titrating amounts at 37°C before being exposed to a constant amount of sCD4. Antibodies of the PGT121 family compete with sCD4 to the same extent. D) VRC01 binding to cells expressing JRFL Env on their surface as observed by flow cytometry. PGT123 Fab (pink), b12 Fab (yellow) and 17b Fab (green) were pre-incubated with the cells in titrating amounts at 37°C before being exposed to a constant amount of VRC01. As expected, b12 Fab that targets the CD4 binding site directly competes with VRC01 binding, and 17b Fab, which binds to the co-receptor binding site, shows no competition with VRC01 binding. PGT123 Fab does not significantly compete with VRC01, a CD4 binding site targeted antibody that does not induce conformational changes upon binding [36]. Binding curves are represented by plotting the dimensionless mean fluorescence intensity (MFI) of VRC01 binding as a function of Fab concentration.
(TIF)

Table S1 **Fitting of atomic models into the image reconstruction using the program Molrep** [**69**] **.** The correct enantiomer of the EM reconstruction was determined by independently fitting the crystal structure of the gp120 trimer (PDB ID 3DNN) and the PGT122 Fab monomers with 3-fold non-crystallographic restraints into the image reconstruction. The correlation coefficients scores reported are from the program Molrep.
(DOCX)

## References

[1] Kwong PD, Mascola JR, Nabel GJ (2009) Mining the B cell repertoire for broadly neutralizing monoclonal antibodies to HIV-1. Cell Host Microbe 6: 292–294. 19837366 10.1016/j.chom.2009.09.008 PMC8513376

[2] Mouquet H, Klein F, Scheid JF, Warncke M, Pietzsch J, et al (2011) Memory B cell antibodies to HIV-1 gp140 cloned from individuals infected with clade A and B viruses. PLoS One 6: e24078. 21931643 10.1371/journal.pone.0024078 PMC3169578

[3] Scheid JF, Mouquet H, Ueberheide B, Diskin R, Klein F, et al (2011) Sequence and structural convergence of broad and potent HIV antibodies that mimic CD4 binding. Science 333: 1633–1637. 21764753 10.1126/science.1207227 PMC3351836

[4] Simek MD, Rida W, Priddy FH, Pung P, Carrow E, et al (2009) Human immunodeficiency virus type 1 elite neutralizers: individuals with broad and potent neutralizing activity identified by using a high-throughput neutralization assay together with an analytical selection algorithm. J Virol 83: 7337–7348. 19439467 10.1128/JVI.00110-09 PMC2704778

[5] Walker LM, Huber M, Doores KJ, Falkowska E, Pejchal R, et al (2011) Broad neutralization coverage of HIV by multiple highly potent antibodies. Nature 477: 466–470. 21849977 10.1038/nature10373 PMC3393110

[6] Walker LM, Phogat SK, Chan-Hui PY, Wagner D, Phung P, et al (2009) Broad and potent neutralizing antibodies from an African donor reveal a new HIV-1 vaccine target. Science 326: 285–289. 19729618 10.1126/science.1178746 PMC3335270

[7] Wu X, Zhou T, Zhu J, Zhang B, Georgiev I, et al (2011) Focused evolution of HIV-1 neutralizing antibodies revealed by structures and deep sequencing. Science 333: 1593–1602. 21835983 10.1126/science.1207532 PMC3516815

[8] Kwong PD, Mascola JR (2012) Human antibodies that neutralize HIV-1: identification, structures, and B cell ontogenies. Immunity 37: 412–425. 22999947 10.1016/j.immuni.2012.08.012 PMC4706166

[9] Huang J, Ofek G, Laub L, Louder MK, Doria-Rose NA, et al (2012) Broad and potent neutralization of HIV-1 by a gp41-specific human antibody. Nature 491: 406–412. 23151583 10.1038/nature11544 PMC4854285

[10] Wang Y, Keck ZY, Foung SK (2011) Neutralizing antibody response to hepatitis C virus. Viruses 3: 2127–2145. 22163337 10.3390/v3112127 PMC3230844

[11] Ekiert DC, Friesen RH, Bhabha G, Kwaks T, Jongeneelen M, et al (2011) A highly conserved neutralizing epitope on group 2 influenza A viruses. Science 333: 843–850. 21737702 10.1126/science.1204839 PMC3210727

[12] Corti D, Voss J, Gamblin SJ, Codoni G, Macagno A, et al (2011) A neutralizing antibody selected from plasma cells that binds to group 1 and group 2 influenza A hemagglutinins. Science 333: 850–856. 21798894 10.1126/science.1205669

[13] Dreyfus C, Laursen NS, Kwaks T, Zuijdgeest D, Khayat R, et al (2012) Highly conserved protective epitopes on influenza B viruses. Science 337: 1343–1348. 22878502 10.1126/science.1222908 PMC3538841

[14] Doria-Rose NA, Klein RM, Manion MM, O'Dell S, Phogat A, et al (2009) Frequency and phenotype of human immunodeficiency virus envelope-specific B cells from patients with broadly cross-neutralizing antibodies. J Virol 83: 188–199. 18922865 10.1128/JVI.01583-08 PMC2612342

[15] Sather DN, Armann J, Ching LK, Mavrantoni A, Sellhorn G, et al (2009) Factors associated with the development of cross-reactive neutralizing antibodies during human immunodeficiency virus type 1 infection. J Virol 83: 757–769. 18987148 10.1128/JVI.02036-08 PMC2612355

[16] Stamatatos L, Morris L, Burton DR, Mascola JR (2009) Neutralizing antibodies generated during natural HIV-1 infection: good news for an HIV-1 vaccine? Nat Med 15: 866–870. 19525964 10.1038/nm.1949

[17] Walker LM, Simek MD, Priddy F, Gach JS, Wagner D, et al (2010) A limited number of antibody specificities mediate broad and potent serum neutralization in selected HIV-1 infected individuals. PLoS Pathog 6: e1001028. 20700449 10.1371/journal.ppat.1001028 PMC2916884

[18] Overbaugh J, Morris L (2012) The antibody response against HIV-1. Cold Spring Harb Perspect Med 2: a007039. 22315717 10.1101/cshperspect.a007039 PMC3253031

[19] Gray ES, Madiga MC, Hermanus T, Moore PL, Wibmer CK, et al (2011) The neutralization breadth of HIV-1 develops incrementally over four years and is associated with CD4+ T cell decline and high viral load during acute infection. J Virol 85: 4828–4840. 21389135 10.1128/JVI.00198-11 PMC3126191

[20] Klein F, Gaebler C, Mouquet H, Sather DN, Lehmann C, et al (2012) Broad neutralization by a combination of antibodies recognizing the CD4 binding site and a new conformational epitope on the HIV-1 envelope protein. J Exp Med 209: 1469–1479. 22826297 10.1084/jem.20120423 PMC3409500

[21] Zhou T, Georgiev I, Wu X, Yang ZY, Dai K, et al (2010) Structural basis for broad and potent neutralization of HIV-1 by antibody VRC01. Science 329: 811–817. 20616231 10.1126/science.1192819 PMC2981354

[22] Bonsignori M, Hwang KK, Chen X, Tsao CY, Morris L, et al (2011) Analysis of a clonal lineage of HIV-1 envelope V2/V3 conformational epitope-specific broadly neutralizing antibodies and their inferred unmutated common ancestors. J Virol 85: 9998–10009. 21795340 10.1128/JVI.05045-11 PMC3196428

[23] Bonomelli C, Doores KJ, Dunlop DC, Thaney V, Dwek RA, et al (2011) The glycan shield of HIV is predominantly oligomannose independently of production system or viral clade. PLoS One 6: e23521. 21858152 10.1371/journal.pone.0023521 PMC3156772

[24] Doores KJ, Bonomelli C, Harvey DJ, Vasiljevic S, Dwek RA, et al (2010) Envelope glycans of immunodeficiency virions are almost entirely oligomannose antigens. Proc Natl Acad Sci U S A 107: 13800–13805. 20643940 10.1073/pnas.1006498107 PMC2922250

[25] Go EP, Chang Q, Liao HX, Sutherland LL, Alam SM, et al (2009) Glycosylation site-specific analysis of clade C HIV-1 envelope proteins. J Proteome Res 8: 4231–4242. 19610667 10.1021/pr9002728 PMC2756219

[26] Go EP, Hewawasam G, Liao HX, Chen H, Ping LH, et al (2011) Characterization of glycosylation profiles of HIV-1 transmitted/founder envelopes by mass spectrometry. J Virol 85: 8270–8284. 21653661 10.1128/JVI.05053-11 PMC3147976

[27] Scanlan CN, Pantophlet R, Wormald MR, Ollmann Saphire E, Stanfield R, et al (2002) The broadly neutralizing anti-human immunodeficiency virus type 1 antibody 2G12 recognizes a cluster of alpha1→2 mannose residues on the outer face of gp120. J Virol 76: 7306–7321. 12072529 10.1128/JVI.76.14.7306-7321.2002 PMC136327

[28] Calarese DA, Scanlan CN, Zwick MB, Deechongkit S, Mimura Y, et al (2003) Antibody domain exchange is an immunological solution to carbohydrate cluster recognition. Science 300: 2065–2071. 12829775 10.1126/science.1083182

[29] Pejchal R, Doores KJ, Walker LM, Khayat R, Huang PS, et al (2011) A potent and broad neutralizing antibody recognizes and penetrates the HIV glycan shield. Science 334: 1097–1103. 21998254 10.1126/science.1213256 PMC3280215

[30] Mouquet H, Scharf L, Euler Z, Liu Y, Eden C, et al (2012) Complex-type N-glycan recognition by potent broadly neutralizing HIV antibodies. Proc Natl Acad Sci U S A 109: E3268–3277. 23115339 10.1073/pnas.1217207109 PMC3511153

[31] Krissinel E, Henrick K (2007) Inference of macromolecular assemblies from crystalline state. J Mol Biol 372: 774–797. 17681537 10.1016/j.jmb.2007.05.022

[32] Depetris RS, Julien JP, Khayat R, Lee JH, Pejchal R, et al (2012) Partial enzymatic deglycosylation preserves the structure of cleaved recombinant HIV-1 envelope glycoprotein trimers. J Biol Chem 287: 24239–24254. 22645128 10.1074/jbc.M112.371898 PMC3397850

[33] Sanders RW, Vesanen M, Schuelke N, Master A, Schiffner L, et al (2002) Stabilization of the soluble, cleaved, trimeric form of the envelope glycoprotein complex of human immunodeficiency virus type 1. J Virol 76: 8875–8889. 12163607 10.1128/JVI.76.17.8875-8889.2002 PMC136973

[34] Stanfield RL, Zemla A, Wilson IA, Rupp B (2006) Antibody elbow angles are influenced by their light chain class. J Mol Biol 357: 1566–1574. 16497332 10.1016/j.jmb.2006.01.023

[35] Liu J, Bartesaghi A, Borgnia MJ, Sapiro G, Subramaniam S (2008) Molecular architecture of native HIV-1 gp120 trimers. Nature 455: 109–113. 18668044 10.1038/nature07159 PMC2610422

[36] Falkowska E, Ramos A, Feng Y, Zhou T, Moquin S, et al (2012) PGV04, an HIV-1 gp120 CD4 binding site antibody, is broad and potent in neutralization but does not induce conformational changes characteristic of CD4. J Virol 86: 4394–4403. 22345481 10.1128/JVI.06973-11 PMC3318667

[37] Madani N, Schon A, Princiotto AM, Lalonde JM, Courter JR, et al (2008) Small-molecule CD4 mimics interact with a highly conserved pocket on HIV-1 gp120. Structure 16: 1689–1701. 19000821 10.1016/j.str.2008.09.005 PMC2597202

[38] Zhao Q, Ma L, Jiang S, Lu H, Liu S, et al (2005) Identification of N-phenyl-N′-(2,2,6,6-tetramethyl-piperidin-4-yl)-oxalamides as a new class of HIV-1 entry inhibitors that prevent gp120 binding to CD4. Virology 339: 213–225. 15996703 10.1016/j.virol.2005.06.008

[39] Kwon YD, Finzi A, Wu X, Dogo-Isonagie C, Lee LK, et al (2012) Unliganded HIV-1 gp120 core structures assume the CD4-bound conformation with regulation by quaternary interactions and variable loops. Proc Natl Acad Sci U S A 109: 5663–5668. 22451932 10.1073/pnas.1112391109 PMC3326499

[40] Moore JP, McKeating JA, Huang YX, Ashkenazi A, Ho DD (1992) Virions of primary human immunodeficiency virus type 1 isolates resistant to soluble CD4 (sCD4) neutralization differ in sCD4 binding and glycoprotein gp120 retention from sCD4-sensitive isolates. J Virol 66: 235–243. 1727487 10.1128/jvi.66.1.235-243.1992 PMC238280

[41] Mulligan MJ, Ritter GD Jr, Chaikin MA, Yamshchikov GV, Kumar P, et al (1992) Human immunodeficiency virus type 2 envelope glycoprotein: differential CD4 interactions of soluble gp120 versus the assembled envelope complex. Virology 187: 233–241. 1736526 10.1016/0042-6822(92)90311-c

[42] Kim M, Chen B, Hussey RE, Chishti Y, Montefiori D, et al (2001) The stoichiometry of trimeric SIV glycoprotein interaction with CD4 differs from that of anti-envelope antibody Fab fragments. J Biol Chem 276: 42667–42676. 11544255 10.1074/jbc.M104166200

[43] Kovacs JM, Nkolola JP, Peng H, Cheung A, Perry J, et al (2012) HIV-1 envelope trimer elicits more potent neutralizing antibody responses than monomeric gp120. Proc Natl Acad Sci U S A 109: 12111–12116. 22773820 10.1073/pnas.1204533109 PMC3409750

[44] Dey B, Svehla K, Xu L, Wycuff D, Zhou T, et al (2009) Structure-based stabilization of HIV-1 gp120 enhances humoral immune responses to the induced co-receptor binding site. PLoS Pathog 5: e1000445. 19478876 10.1371/journal.ppat.1000445 PMC2680979

[45] McLellan JS, Pancera M, Carrico C, Gorman J, Julien JP, et al (2011) Structure of HIV-1 gp120 V1/V2 domain with broadly neutralizing antibody PG9. Nature 23: 336–343. 10.1038/nature10696 PMC3406929 22113616

[46] Platt EJ, Gomes MM, Kabat D (2012) Kinetic mechanism for HIV-1 neutralization by antibody 2G12 entails reversible glycan binding that slows cell entry. Proc Natl Acad Sci U S A 109: 7829–7834. 22547820 10.1073/pnas.1109728109 PMC3356642

[47] Trkola A, Dragic T, Arthos J, Binley JM, Olson WC, et al (1996) CD4-dependent, antibody-sensitive interactions between HIV-1 and its co-receptor CCR-5. Nature 384: 184–187. 8906796 10.1038/384184a0

[48] Garlick RL, Kirschner RJ, Eckenrode FM, Tarpley WG, Tomich CS (1990) *Escherichia coli* expression, purification, and biological activity of a truncated soluble CD4. AIDS Res Hum Retroviruses 6: 465–479. 2187501 10.1089/aid.1990.6.465

[49] Pejchal R, Walker LM, Stanfield RL, Phogat SK, Koff WC, et al (2010) Structure and function of broadly reactive antibody PG16 reveal an H3 subdomain that mediates potent neutralization of HIV-1. Proc Natl Acad Sci U S A 107: 11483–11488. 20534513 10.1073/pnas.1004600107 PMC2895122

[50] Klock HE, Lesley SA (2009) The Polymerase Incomplete Primer Extension (PIPE) method applied to high-throughput cloning and site-directed mutagenesis. Methods Mol Biol 498: 91–103. 18988020 10.1007/978-1-59745-196-3_6

[51] Ekiert DC, Kashyap AK, Steel J, Rubrum A, Bhabha G, et al (2012) Cross-neutralization of influenza A viruses mediated by a single antibody loop. Nature 489: 526–532. 22982990 10.1038/nature11414 PMC3538848

[52] Kabsch W (2010) Xds. Acta Crystallogr D Biol Crystallogr 66: 125–132. 20124692 10.1107/S0907444909047337 PMC2815665

[53] McCoy AJ, Grosse-Kunstleve RW, Adams PD, Winn MD, Storoni LC, et al (2007) Phaser crystallographic software. J Appl Crystallogr 40: 658–674. 19461840 10.1107/S0021889807021206 PMC2483472

[54] Brunger AT, Adams PD, Clore GM, DeLano WL, Gros P, et al (1998) Crystallography & NMR system: A new software suite for macromolecular structure determination. Acta Crystallogr D Biol Crystallogr 54: 905–921. 9757107 10.1107/s0907444998003254

[55] Winn MD, Ballard CC, Cowtan KD, Dodson EJ, Emsley P, et al (2011) Overview of the CCP4 suite and current developments. Acta Crystallogr D Biol Crystallogr 67: 235–242. 21460441 10.1107/S0907444910045749 PMC3069738

[56] Adams PD, Afonine PV, Bunkoczi G, Chen VB, Davis IW, et al (2010) PHENIX: a comprehensive Python-based system for macromolecular structure solution. Acta Crystallogr D Biol Crystallogr 66: 213–221. 20124702 10.1107/S0907444909052925 PMC2815670

[57] Emsley P, Cowtan K (2004) Coot: model-building tools for molecular graphics. Acta Crystallogr D Biol Crystallogr 60: 2126–2132. 15572765 10.1107/S0907444904019158

[58] Li M, Gao F, Mascola JR, Stamatatos L, Polonis VR, et al (2005) Human immunodeficiency virus type 1 env clones from acute and early subtype B infections for standardized assessments of vaccine-elicited neutralizing antibodies. J Virol 79: 10108–10125. 16051804 10.1128/JVI.79.16.10108-10125.2005 PMC1182643

[59] Doores KJ, Burton DR (2010) Variable loop glycan dependency of the broad and potent HIV-1-neutralizing antibodies PG9 and PG16. J Virol 84: 10510–10521. 20686044 10.1128/JVI.00552-10 PMC2950566

[60] Blixt O, Head S, Mondala T, Scanlan C, Huflejt ME, et al (2004) Printed covalent glycan array for ligand profiling of diverse glycan binding proteins. Proc Natl Acad Sci U S A 101: 17033–17038. 15563589 10.1073/pnas.0407902101 PMC534418

[61] Xu R, McBride R, Nycholat CM, Paulson JC, Wilson IA (2012) Structural characterization of the hemagglutinin receptor specificity from the 2009 H1N1 influenza pandemic. J Virol 86: 982–990. 22072785 10.1128/JVI.06322-11 PMC3255799

[62] Nycholat CM, McBride R, Ekiert DC, Xu R, Rangarajan J, et al (2012) Recognition of sialylated poly-N-acetyllactosamine chains on N- and O-linked glycans by human and avian influenza A virus hemagglutinins. Angew Chem Int Ed Engl 51: 4860–4863. 22505324 10.1002/anie.201200596 PMC3517101

[63] Suloway C, Pulokas J, Fellmann D, Cheng A, Guerra F, et al (2005) Automated molecular microscopy: the new Leginon system. J Struct Biol 151: 41–60. 15890530 10.1016/j.jsb.2005.03.010

[64] Voss NR, Yoshioka CK, Radermacher M, Potter CS, Carragher B (2009) DoG Picker and TiltPicker: software tools to facilitate particle selection in single particle electron microscopy. J Struct Biol 166: 205–213. 19374019 10.1016/j.jsb.2009.01.004 PMC2768396

[65] Mindell JA, Grigorieff N (2003) Accurate determination of local defocus and specimen tilt in electron microscopy. J Struct Biol 142: 334–347. 12781660 10.1016/s1047-8477(03)00069-8

[66] Hohn M, Tang G, Goodyear G, Baldwin PR, Huang Z, et al (2007) SPARX, a new environment for Cryo-EM image processing. J Struct Biol 157: 47–55. 16931051 10.1016/j.jsb.2006.07.003

[67] Tang G, Peng L, Baldwin PR, Mann DS, Jiang W, et al (2007) EMAN2: an extensible image processing suite for electron microscopy. J Struct Biol 157: 38–46. 16859925 10.1016/j.jsb.2006.05.009

[68] Sorzano CO, Bilbao-Castro JR, Shkolnisky Y, Alcorlo M, Melero R, et al (2010) A clustering approach to multireference alignment of single-particle projections in electron microscopy. J Struct Biol 171: 197–206. 20362059 10.1016/j.jsb.2010.03.011 PMC2893300

[69] Vagin A, Teplyakov A (2010) Molecular replacement with MOLREP. Acta Crystallogr D Biol Crystallogr 66: 22–25. 20057045 10.1107/S0907444909042589

[70] Pettersen EF, Goddard TD, Huang CC, Couch GS, Greenblatt DM, et al (2004) UCSF Chimera–a visualization system for exploratory research and analysis. J Comput Chem 25: 1605–1612. 15264254 10.1002/jcc.20084

[71] Gasteiger EHC, Gattiker A, Duvaud S, Wilkins MR, Appel RD, Bairoch A (2005) Protein Identification and Analysis Tools on the ExPASy Server. In: The Proteomics Protocols Handbook Walker JM, editor. Humana Press 571–607.

[72] Waterhouse AM, Procter JB, Martin DM, Clamp M, Barton GJ (2009) Jalview Version 2–a multiple sequence alignment editor and analysis workbench. Bioinformatics 25: 1189–1191. 19151095 10.1093/bioinformatics/btp033 PMC2672624

[73] Matthews BW (1968) Solvent content of protein crystals. J Mol Biol 33: 491–497. 5700707 10.1016/0022-2836(68)90205-2

[74] Kantardjieff KA, Rupp B (2003) Matthews coefficient probabilities: Improved estimates for unit cell contents of proteins, DNA, and protein-nucleic acid complex crystals. Protein Sci 12: 1865–1871. 12930986 10.1110/ps.0350503 PMC2323984

[75] de Azevedo WF Jr, Dias R (2008) Experimental approaches to evaluate the thermodynamics of protein-drug interactions. Curr Drug Targets 9: 1071–1076. 19128217 10.2174/138945008786949441

