# Supplementary figures and images for: Broadly Neutralizing Antibody PGT121 Allosterically Modulates CD4 Binding via Recognition of the HIV-1 gp120 V3 Base and Multiple Surrounding Glycans
Source: PLoS Pathog. 2013 May 2;9(5):e1003342. doi: 10.1371/journal.ppat.1003342 (PMC3642082; doi:10.1371/journal.ppat.1003342)

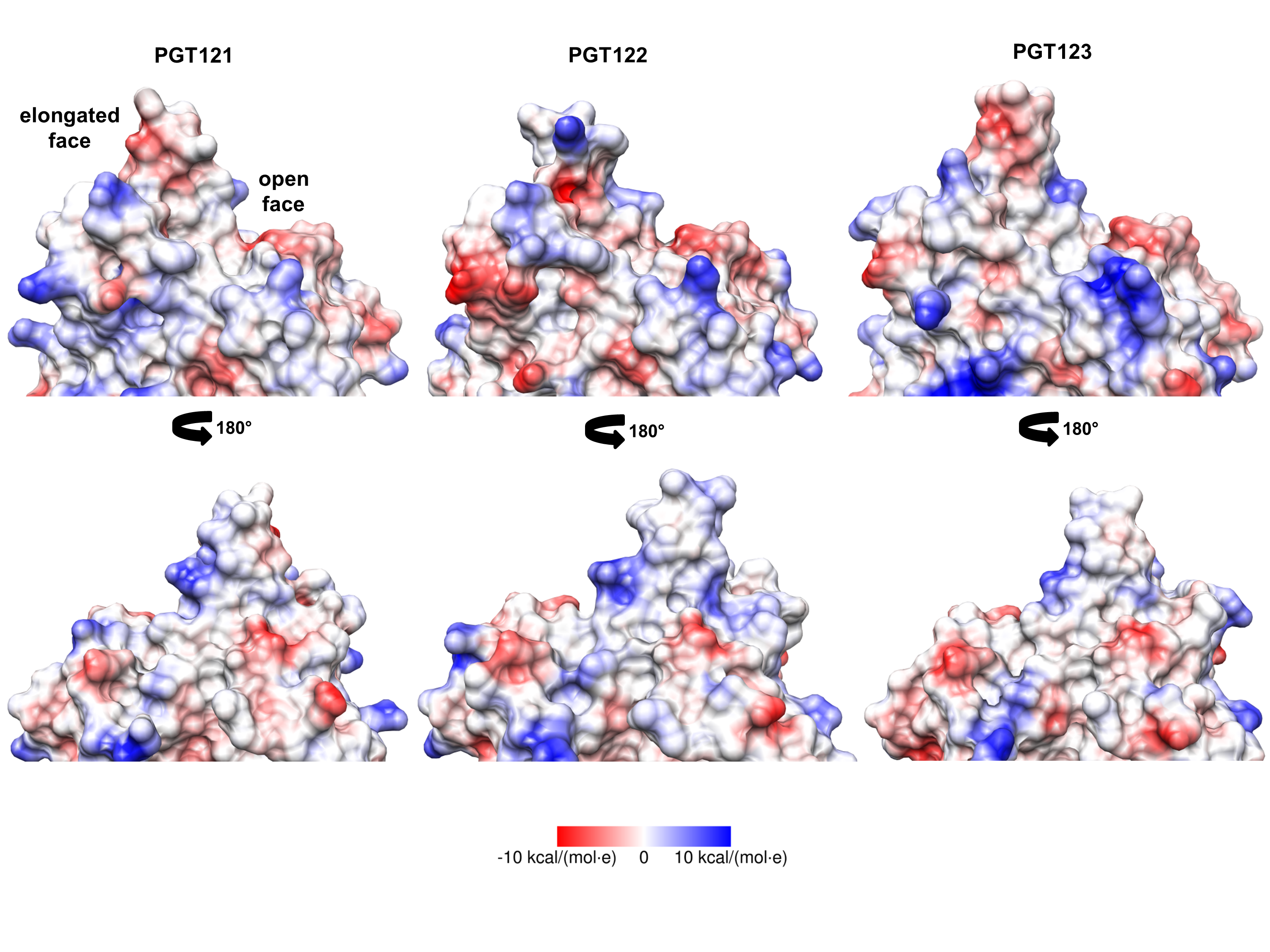

Supplement: Figure S1 — Electrostatic rendering of the paratope of PGT121 antibodies. Surfaces are colored according to their electrostatic potential, with red, blue and white surfaces representing regions of negative, positive and neutral electrostatic potential, respectively. Overall, the elongated and open faces of the PGT121 antibodies have similar electrostatic potential properties, particularly in the conserved regions detailed in Fig. 1B. Only the antibody Fv region is shown for clarity. This figure was generated using UCSF Chimera [70]. (TIF) [file ppat.1003342.s001.tif]

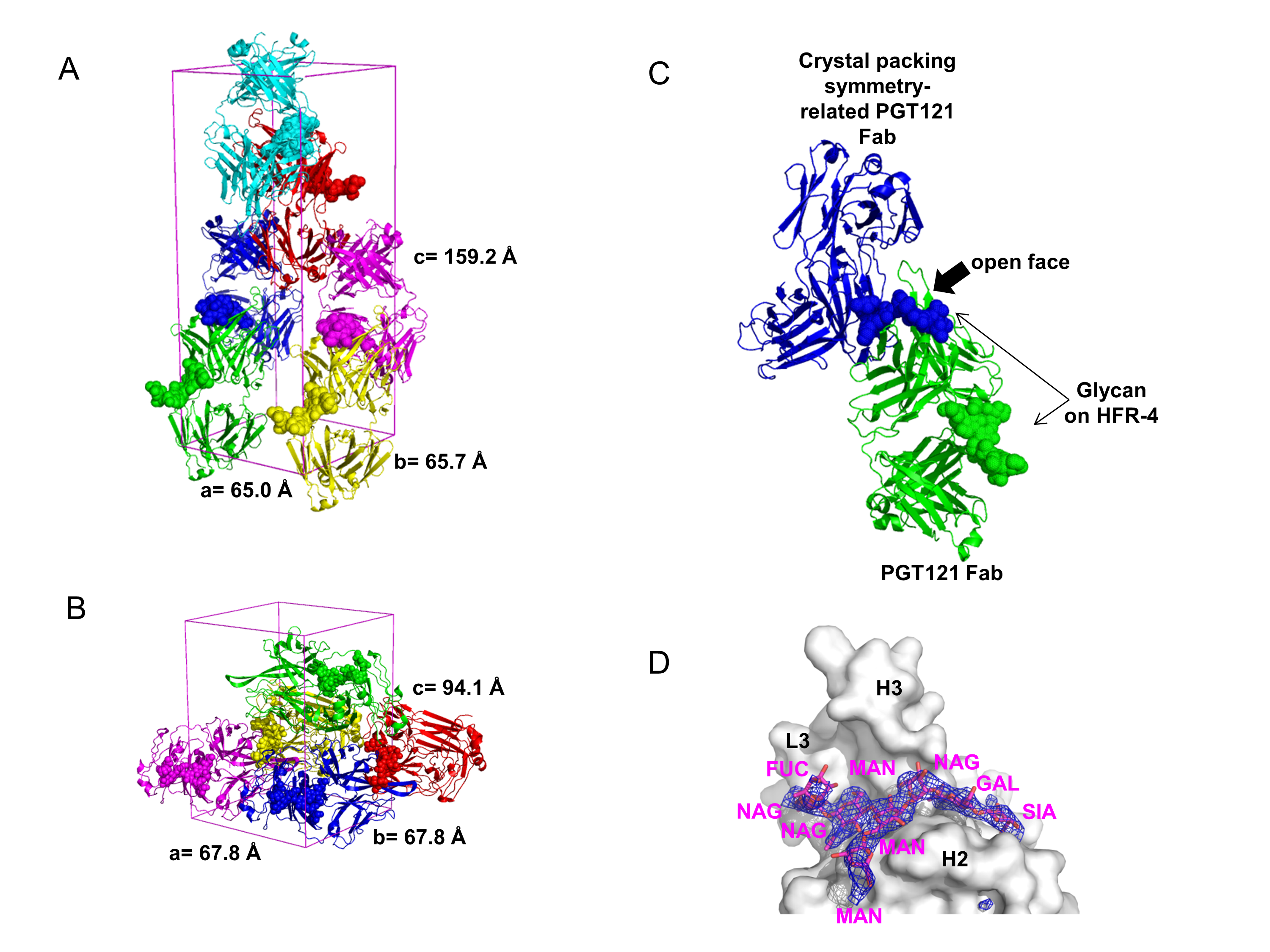

Supplement: Figure S2 — The crystal packing of the PGT121 antibody structure results in a complex glycan located in the antibody paratope. A) Assembly of PGT121 Fab in the crystal lattice in space group P212121 with unit cell dimensions of a = 65.0 Å, b = 65.7 Å, c = 159.2 Å (magenta box). Calculations of the Matthews' coefficient indicate a Vm = 3.59 Å3/Da and 65.8% solvent content with one molecule per asymmetric unit [73], [74]. The PGT121 Fab is rendered as secondary structure cartoon with symmetry-related molecules colored differently. The glycan on PGT121 heavy chain framework 4 (HFR-4) is shown as spheres. B. Assembly of PGT121 Fab in the crystal lattice in space group P212121 with unit cell dimensions of a = 67.8 Å, b = 67.8 Å, c = 94.1 Å (magenta box), PDB ID 4FQC [30]. Calculations of the Matthews coefficient indicate a Vm = 2.29 Å3/Da and 46.2% solvent content with one molecule per asymmetric unit [73], [74]. The PGT121 Fab is rendered as secondary structure cartoon with symmetry-related molecules colored differently. The glycan on PGT121 HFR-4 is shown as spheres. Although the crystal packing in this system is significantly different than in A), it leads to the glycan moiety sitting in the same paratope region in both structures. C) As in PDB ID 4FQC, the crystal packing in the PGT121 structure reported here puts the symmetry-related biantennary carbohydrate from the heavy chain FR-4 in the paratope open face. This fortuitous interaction in the crystal allows an appreciation for understanding how the PGT121 paratope can accommodate glycan moieties in its paratope. D) Another view from that shown in Fig. 2B of the PGT121 open face binding to a biantennary glycan from a symmetry-related molecule in the crystal lattice. Rendering of PGT121 and the glycan is the same as in Fig. 2B. The blue mesh is a 2Fo-Fc electron density map contoured at a 1.2 sigma level around the glycan moiety. Here again, the two N-acetylglucosamines (NAG) that would be attached to the Asn on the protein are [file ppat.1003342.s002.tif]

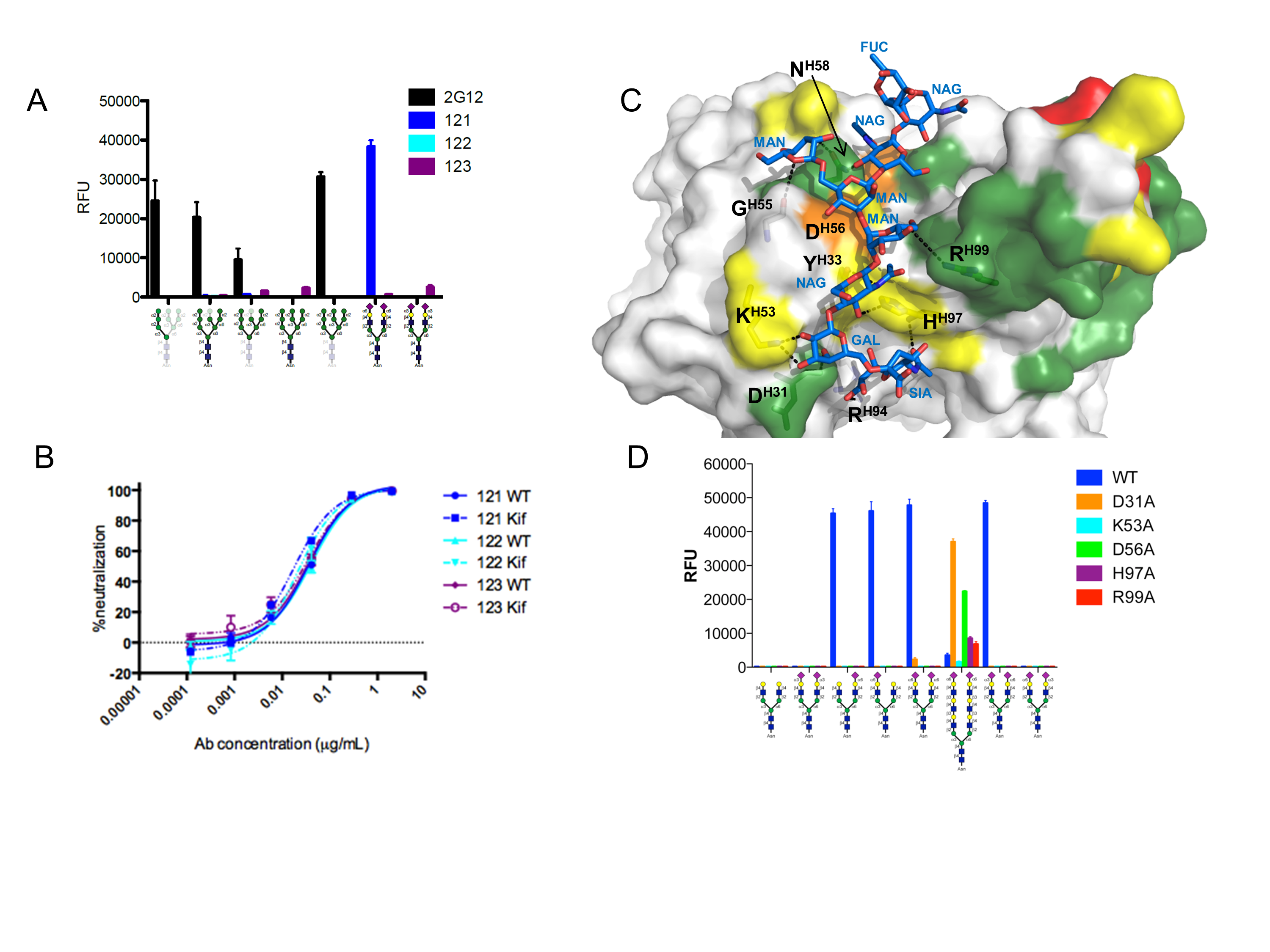

Supplement: Figure S3 — Glycan binding properties of antibodies of the PGT121 family. A) In contrast to antibody 2G12, antibodies of the PGT121 family do not possess high affinity for glycan moieties representative of oligomannose motifs. PGT121 does, however, interact favorably with biantennary N-linked glycans terminating with one or two α-2–6 linked sialic acids. Thus, from these data, it is not known from which site on the Env trimer this glycan would emanate. Blue squares = N-acetyl glucosamine; green circles = mannose; yellow circles = galactose; pink diamond = sialic acid. RFU = relative fluorescence units. B) Although PGT121 is able to bind a complex sugar on the glycan array, it is able to neutralize JRFL pseudovirus made in the presence of kifunensine with a similar potency. Kifunensine-treated pseudovirus displays mainly Man9GlcNAc2 glycans and suggests that PGT121 reactivity with a biantennary complex sugar is not required for HIV-1 neutralization. C) Top view of the PGT121 paratope, color-coded by the importance of paratope residues on HIV-1 neutralization, as in Fig. 2. The observed biantennary glycan sits in the secondary “open-face” paratope groove, and hydrogen bonds are mediated by side-chain and backbone atoms of residues DH31, YH33, KH53, GH55, NH58, RH94, HH97 and RH99. Of the hydrogen-bonding residues, only YH33, KH53 and HH97 were identified by alanine-scanning mutagenesis as being moderately important in mediating HIV-1 neutralization. D) Alanine mutants of PGT121 paratope residues forming the glycan binding pocket knock out glycan binding, confirming that the glycan observed in the crystal structure is representative of binding to biantennary N-linked glycans terminated in 2–6 linked sialic acids in the glycan array. Particularly, the KH53A mutation abrogates binding to all glycan types. Lysine at position KH53 is only present in PGT121, and not in PGT122 and PGT123, possibly helping to explain the higher reactivity of PGT121 on the glycan array when compared to PG [file ppat.1003342.s003.tif]

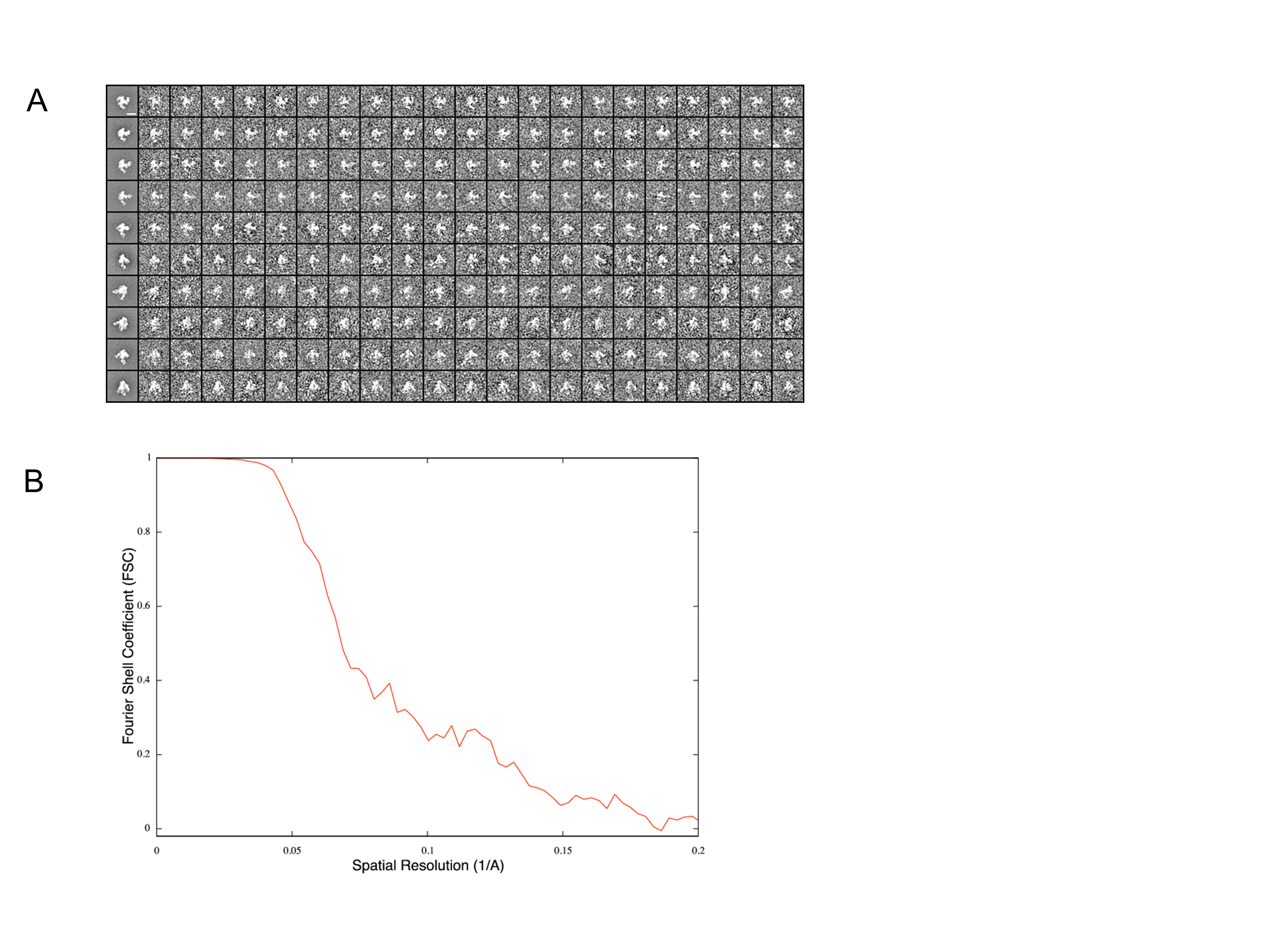

Supplement: Figure S4 — Negative stain electron microscopy of the SOSIP.664:PGT122 Fab complex. A) Reference free class averages of the SOSIP.664:PGT122 Fab complex calculated from 60 particle orientations are shown on the far left. Representative raw particles for each class average, low pass filtered to 15 Å resolution are shown at the right. The white bar in the first box on the upper left corresponds to ∼100 Å. B. Fourier shell correlation (FSC) curve of the SOSIP.664:PGT122 Fab complex image reconstruction. The curve measures the correlation between two independent image reconstructions as calculated from two halves of the entire data set (10,413 particles). The resolution of the image reconstruction is measured to be ∼15 Å resolution based on an FSC of 0.5. (TIF) [file ppat.1003342.s004.tif]

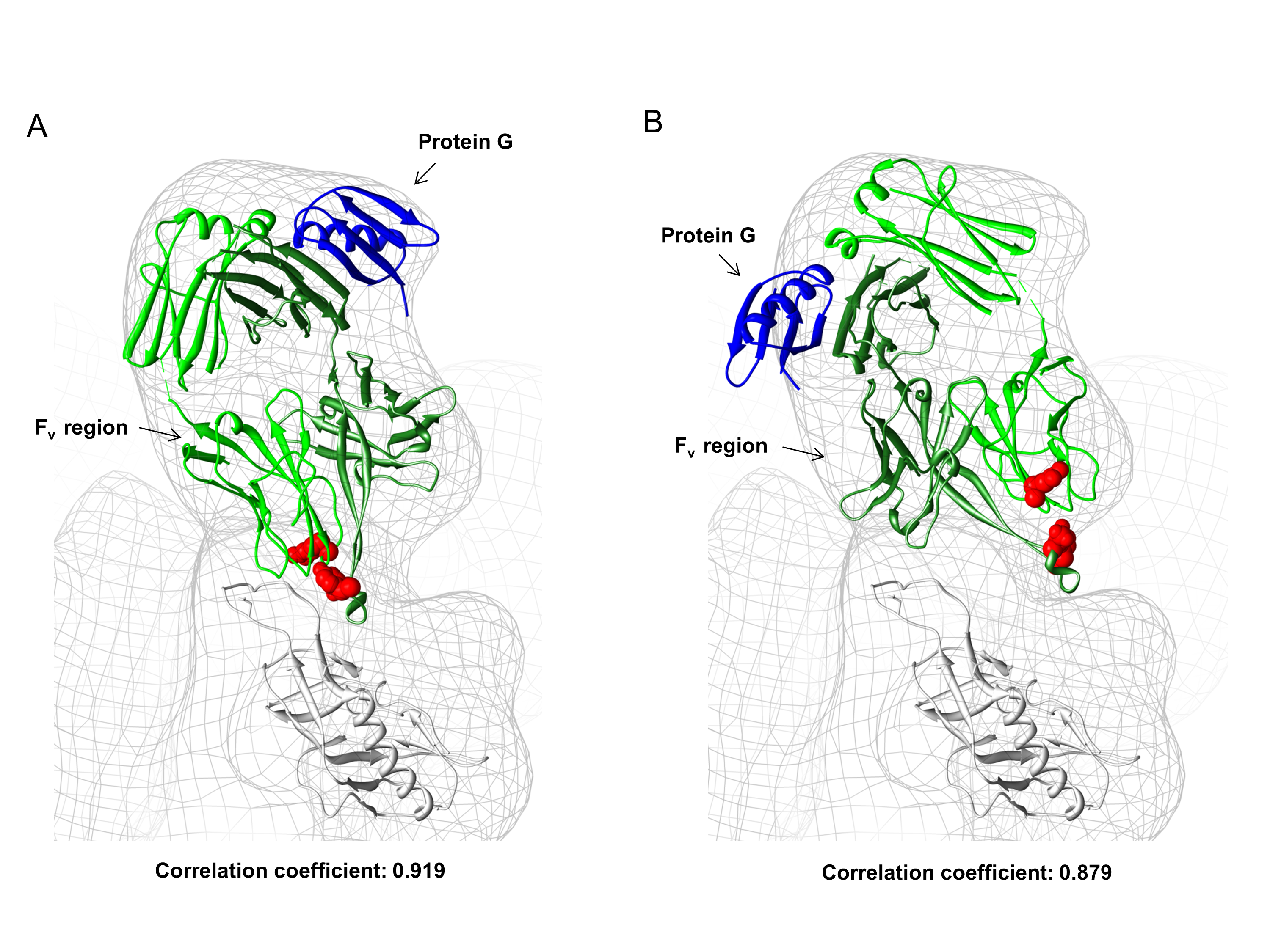

Supplement: Figure S5 — Fitting of the PGT122 Fab in the EM reconstruction of the SOSIP.664:PGT122 Fab+Protein G complex. To ascertain the correct fitting of the PGT122 Fab from the two possible orientations in the EM reconstruction, Protein G was added to correctly position the CH1 fragment. The PGT122 Fab crystal structure (light and heavy chains are colored in light and dark green, respectively) and the eODmV3 crystal structure (PDB ID 3TYG [29]) (colored in gray) were fitted using the “Fit in map” tool in the negative-stain EM reconstruction (transparent gray mesh) using UCSF Chimera [70]. The Protein G - PGT122 Fab interaction was modeled according to the previously solved crystal structure of a mouse Fab in complex with Protein G (PDB ID 1IGC). The crystal structures are rendered as secondary structure cartoons. The model presented in A) most likely represents the correct orientation of PGT122 Fab binding to HIV-1 Env gp140 because of 1) an excellent fit of the Protein G model in the density; 2) an excellent fit of the Fv region; and 3) residues identified by alanine scanning mutagenesis as critical for mediating HIV-1 neutralization (red spheres) fall inside the EM density and point toward gp120 elements. These key requirements are not met in the alternate model presented in B). The higher correlation coefficient for model A) is indicative of the attributes mentioned above. (TIF) [file ppat.1003342.s005.tif]

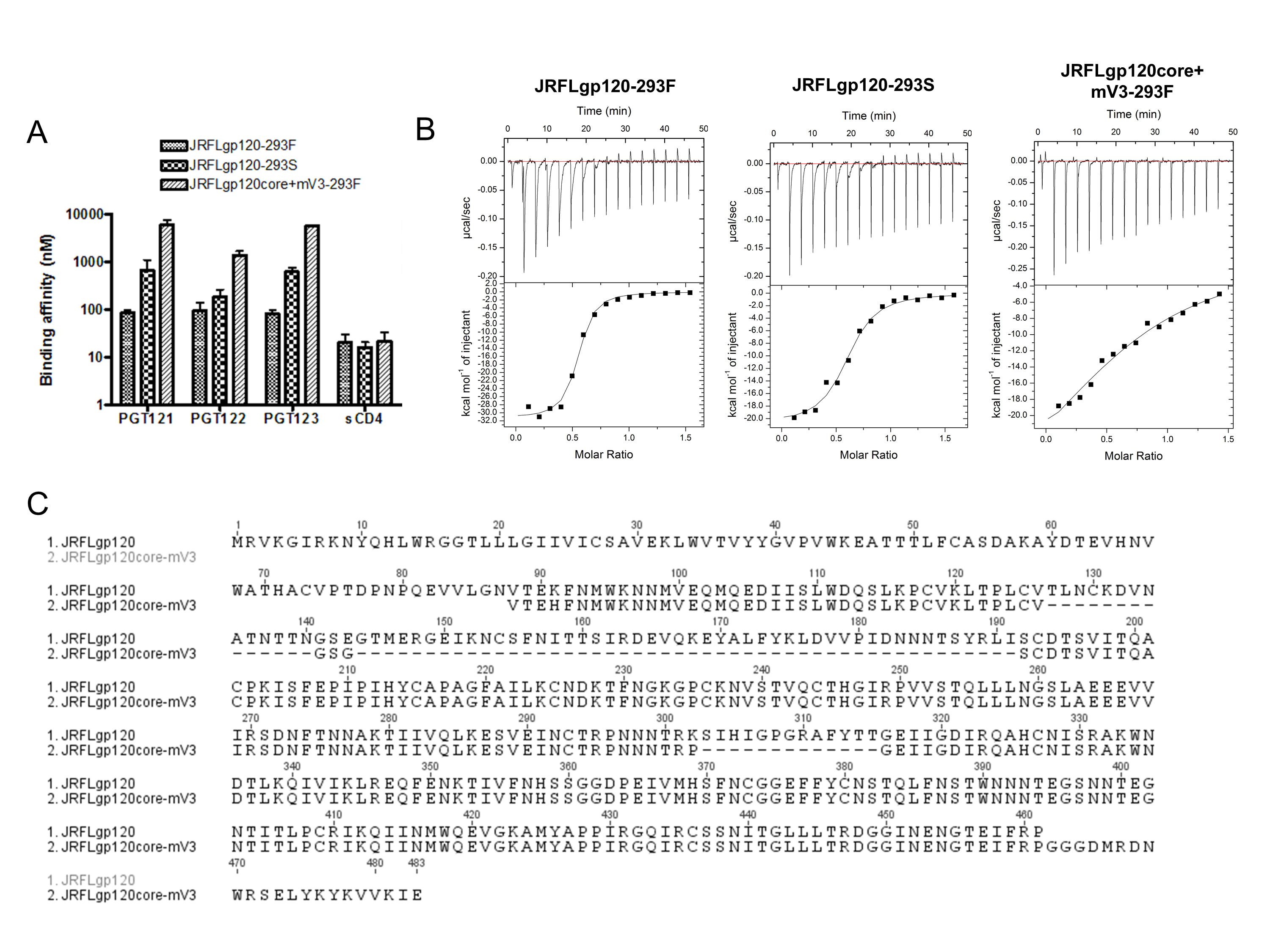

Supplement: Figure S6 — Binding of PGT121 antibodies to different gp120 constructs, as evaluated in ITC experiments. A) Bar graph showing the binding affinity (Kd) of PGT121 antibodies for three different gp120 monomeric constructs. Binding of sCD4 was used as a control. A maximum binding affinity of ∼80 nM is observed for all three PGT121 antibodies. Expression in a cell-line leading to immature oligomannose glycans (HEK 293S cells) reduces the affinity moderately, whereas deletion of C1, V1/V2 and V3 tip results in a 10–100 fold decrease in binding affinity. B) Representative ITC binding isotherms for the data presented in A). The top panel shows representative raw data and the bottom panel is the binding isotherm. C) Sequence alignment of the two gp120 monomeric constructs used in the current study. (TIF) [file ppat.1003342.s006.tif]

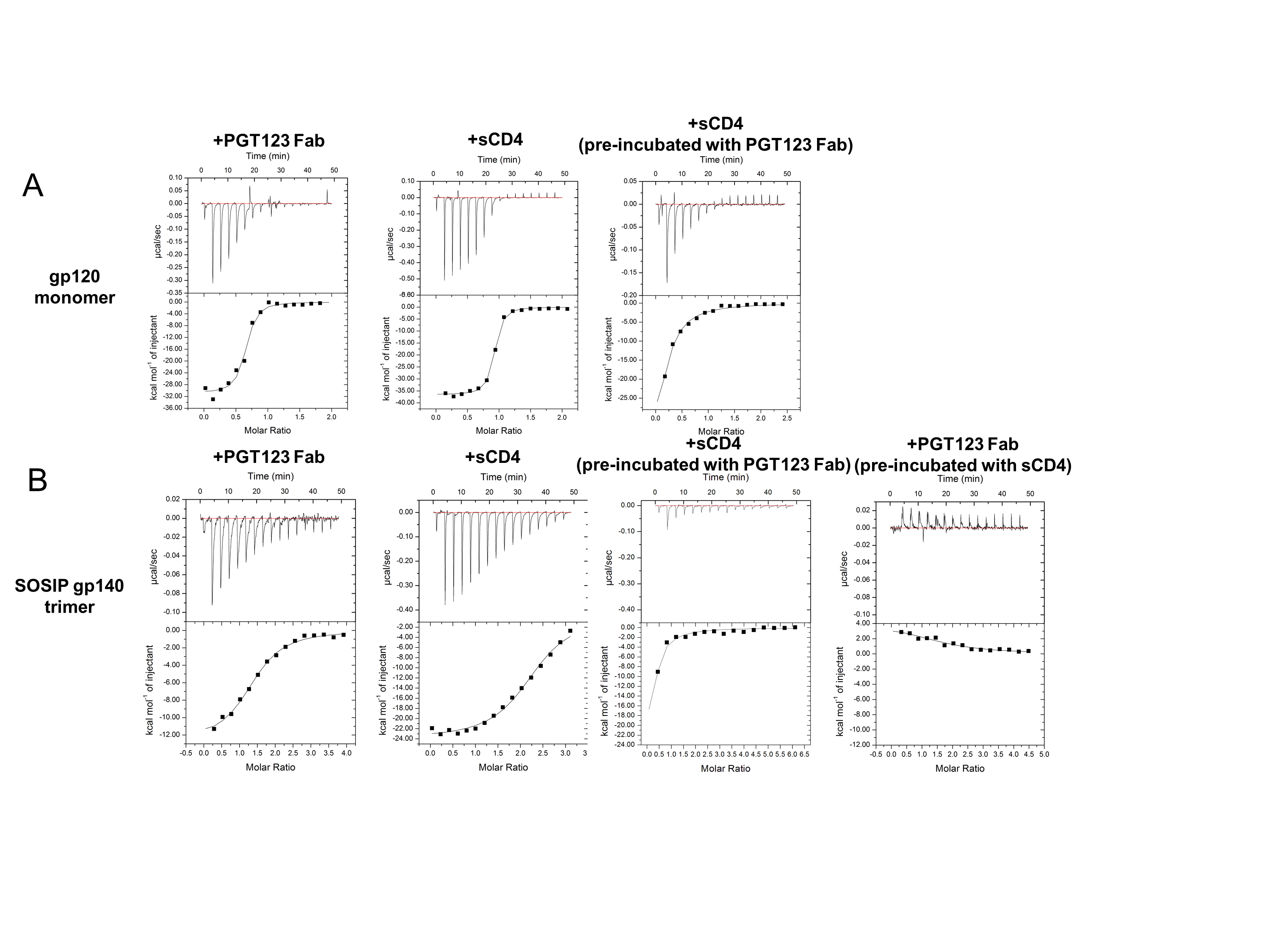

Supplement: Figure S7 — Competition between PGT123 and sCD4 for binding to monomeric gp120 and SOSIP.664 gp140 trimers, as evaluated in ITC experiments. The data are representative of those obtained with PGT121 and PGT122 antibodies. Top panel shows representative raw data and the bottom panel is the binding isotherm. A) ITC experiments of PGT123 Fab and sCD4 binding to a gp120 monomeric construct. Whereas individual binding experiments reveal high affinity binding (left panel and center panel), pre-incubation of monomeric gp120 with PGT123 leads to a significant loss in binding of sCD4, as observed from a decrease in both binding affinity and stoichiometry (right panel). B) Binding isotherms for mixing of sCD4 into SOSIP.664 (second panel) and into a pre-formed PGT123 Fab:SOSIP.664 complex (third panel). Although sCD4 binds well to the unliganded SOSIP trimer, pre-incubation of the SOSIP trimer with PGT123 Fab almost completely abrogates binding by sCD4, as evidenced by the lack of significant heat produced upon mixing. PGT123 Fab mixing with SOSIP.664 in ITC experiments results in saturating binding (first panel); however, pre-incubation of SOSIP.664 with sCD4 almost completely inhibits PGT123 Fab binding (fourth panel), suggesting that optimal PGT123 interaction with the HIV-1 Env trimer probably occurs prior to CD4 receptor engagement. Binding parameters for all ITC experiments are reported in Table 2. (TIF) [file ppat.1003342.s007.tif]

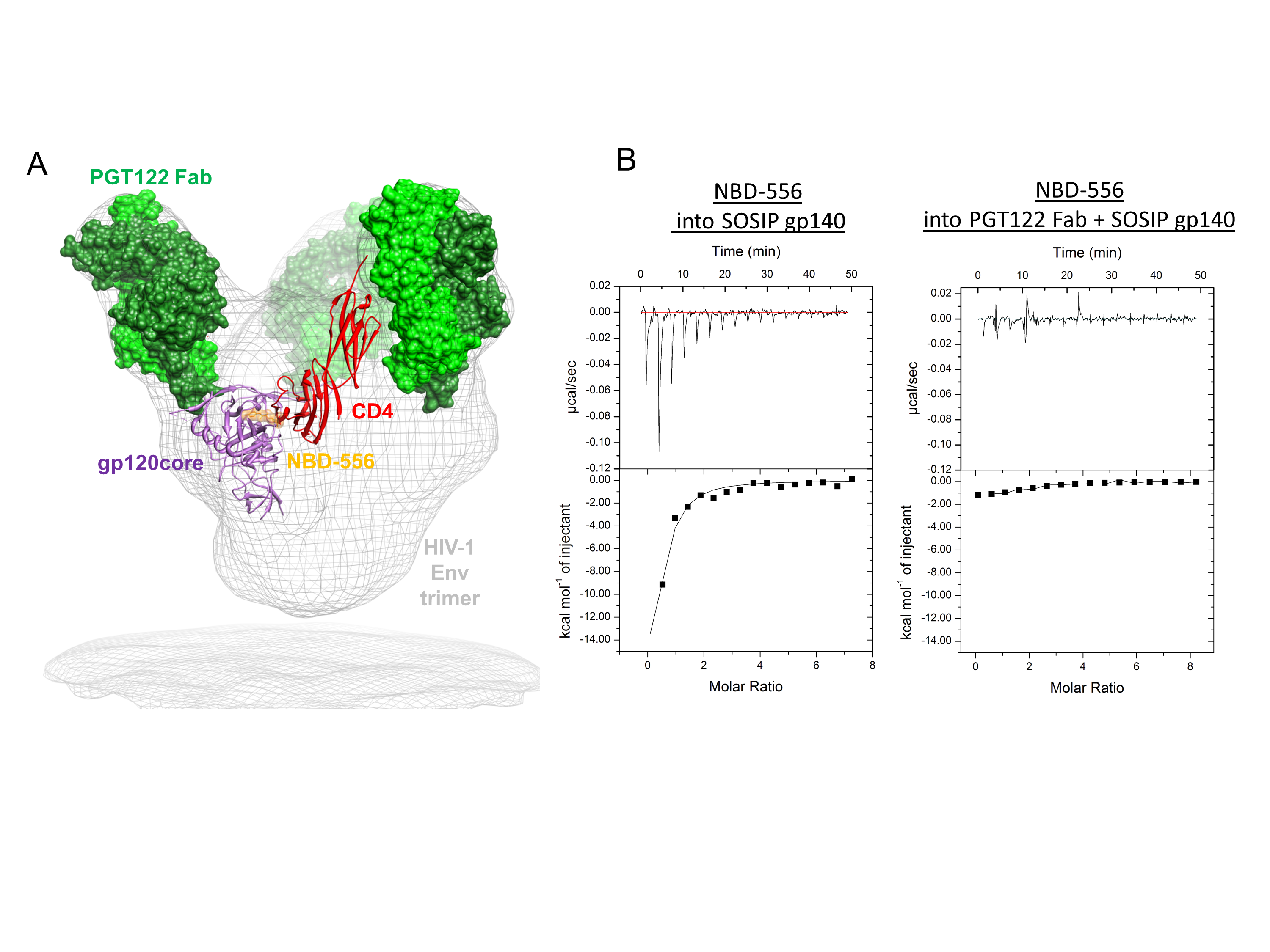

Supplement: Figure S8 — Competition between PGT122 and a small molecule sCD4 mimic, NBD-556, for binding to SOSIP.664 gp140 trimers, as evaluated in ITC experiments. A) Model of PGT122 Fab interacting with the SOSIP.664 gp140 trimer. Rendering is as in Figs. 5 and S5. PGT122 Fab binding does not sterically occlude the CD4 binding site. Thus, the CD4 binding site should remain accessible for binding by sCD4 (red secondary structure cartoon) and a small molecular CD4 mimic of 337.8 Da, NBD-556 (orange sticks and surface). B) Binding isotherms for mixing of NBD-556 into SOSIP.664 (first panel) and into a pre-formed PGT122 Fab:SOSIP.664 complex (second panel). Although NBD-556 binds well to the unliganded SOSIP trimer (Kd = 1.7 µM), pre-incubation of the SOSIP trimer with PGT122 Fab almost completely abrogates binding by NBD-556, as evidenced by the lack of significant heat produced upon mixing. The top panel shows representative raw data and the bottom panel is the binding isotherm. Together, these data support the hypothesis that antibodies of the PGT121 family block interactions with CD4 binding site elements that induce conformational changes, such as sCD4 and NBD-556, by mechanism other than steric occlusion, and possibly through allostery. (TIF) [file ppat.1003342.s008.tif]

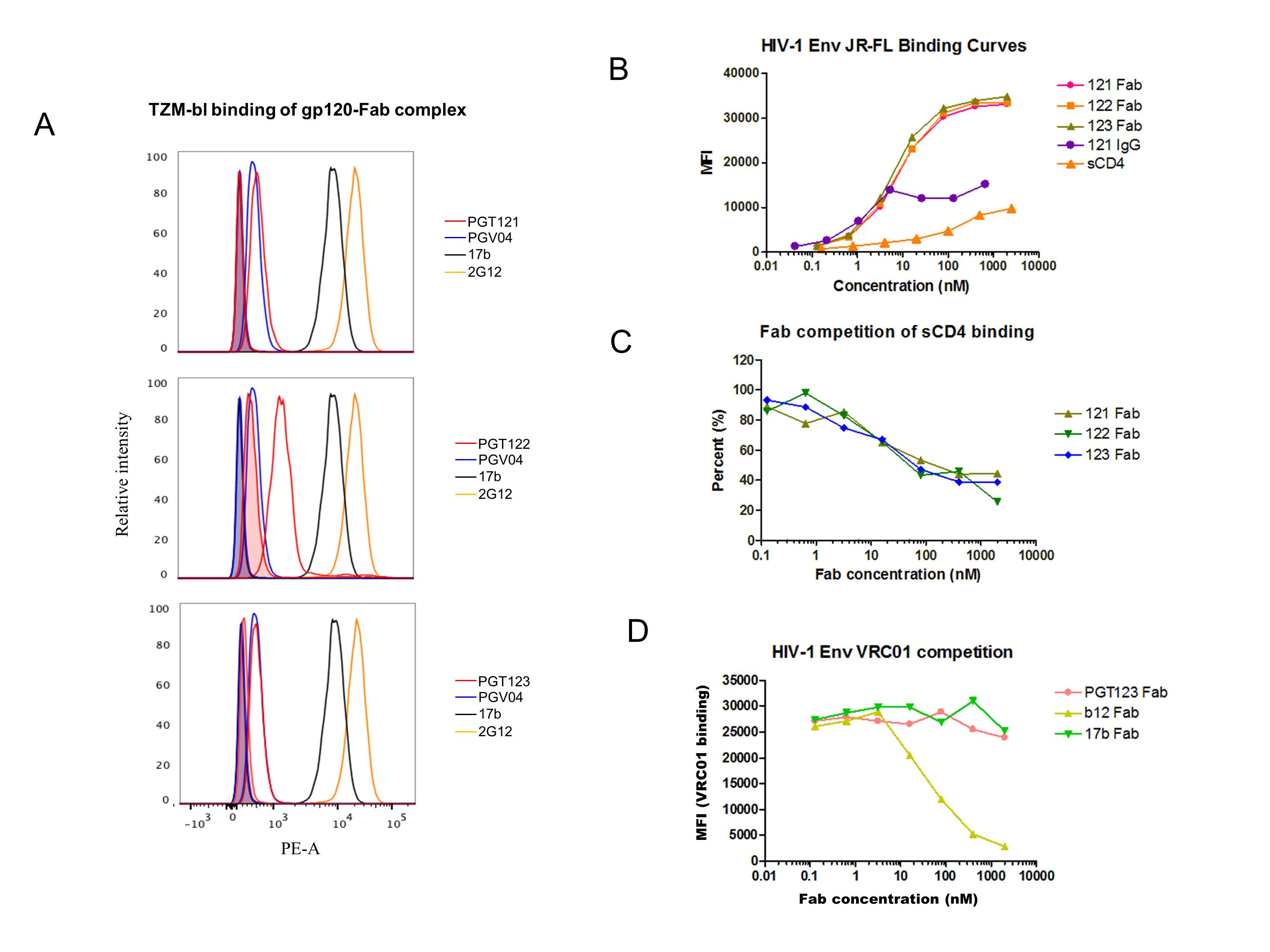

Supplement: Figure S9 — Competition between antibodies of the PGT121 family and CD4 for binding to cell surface components, as determined in FACS experiments. A) SEC-purified complexes of gp120 with various Fabs were tested for their ability to bind CD4+ TZM-bl cells by FACS. Whereas 17b+gp120 (black) and 2G12+gp120 (orange) complexes bound well to CD4+ TZM-bl cells, PGT121+gp120, PGT122+gp120 and PGT123+gp120 complexes (red) were not able to engage CD4+ TZM-bl cells. Lack of binding of the gp120 complexes with PGT121, PGT122 and PGT123 is comparable to that of the CD4-binding site antibody PGV04 in complex with gp120 (blue). On the x-axis, PE-A represents the relative intensity of detected Fab on the surface of CD4+ TZM-bl cells. Filled areas indicate Fab alone (negative control), whereas hollow areas are for the Fab-gp120 complexes. B) Binding curves of elements used in the competition assays to cell-surface HIV-1 JRFL Env. C) sCD4 binding to cells expressing JRFL Env on their surface as observed by flow cytometry. PGT121 (brown), 122 (green) and 123 (blue) Fab were pre-incubated with the cells in titrating amounts at 37°C before being exposed to a constant amount of sCD4. Antibodies of the PGT121 family compete with sCD4 to the same extent. D) VRC01 binding to cells expressing JRFL Env on their surface as observed by flow cytometry. PGT123 Fab (pink), b12 Fab (yellow) and 17b Fab (green) were pre-incubated with the cells in titrating amounts at 37°C before being exposed to a constant amount of VRC01. As expected, b12 Fab that targets the CD4 binding site directly competes with VRC01 binding, and 17b Fab, which binds to the co-receptor binding site, shows no competition with VRC01 binding. PGT123 Fab does not significantly compete with VRC01, a CD4 binding site targeted antibody that does not induce conformational changes upon binding [36]. Binding curves are represented by plotting the dimensionless mean fluorescence intensity (MFI) of VRC01 binding as a function of Fab concentration. ( [file ppat.1003342.s009.tif]
