# Supplementary material for: Broadly Neutralizing Antibody PGT121 Allosterically Modulates CD4 Binding via Recognition of the HIV-1 gp120 V3 Base and Multiple Surrounding Glycans
Source: PLoS Pathog. 2013 May 2;9(5):e1003342. doi: 10.1371/journal.ppat.1003342 (PMC3642082; doi:10.1371/journal.ppat.1003342)
Supplement: Table S1 — Fitting of atomic models into the image reconstruction using the program Molrep [69] . The correct enantiomer of the EM reconstruction was determined by independently fitting the crystal structure of the gp120 trimer (PDB ID 3DNN) and the PGT122 Fab monomers with 3-fold non-crystallographic restraints into the image reconstruction. The correlation coefficients scores reported are from the program Molrep. (DOCX) [file ppat.1003342.s010.docx]

| Model | Enantiomer 1 | Enantiomer 2 |
| --- | --- | --- |
| HIV core (PDB ID 3DNN) | 0.307 | 0.214 |
| PGT122 Fab | 0.400 | 0.239 |
